# Impact of an educational intervention on postpartum depression for primary care nurses: a quasi-experimental study

**DOI:** 10.1590/1980-220X-REEUSP-2025-0032en

**Published:** 2025-09-08

**Authors:** Débora Alves da Silva, Marli Aparecida Reis Coimbra, Lucas Carvalho Santana, Maria Aline Leocádio, Fernanda Bonato Zuffi, Lúcia Aparecida Ferreira

**Affiliations:** 1 Universidade Federal do Triângulo Mineiro, Uberaba, MG, Brazil.; 2 Universidade Federal do Triângulo Mineiro, Hospital de Clínicas, Uberaba, MG, Brazil.

**Keywords:** Nurses, Depression, Postpartum, Education, Continuing, Primary Health Care, Teaching

## Abstract

**Objective:**

To evaluate the impact of an educational intervention on nursing care for women with signs of postpartum depression for primary health care nurses.

**Method:**

Quasi-experimental, before-and-after study carried out with 14 primary health care nurses from a municipality, who participated in an educational intervention on nursing care for women with signs of postpartum depression. Qualitative data analysis was carried out before and after the intervention, using Bardin’s thematic content analysis.

**Results:**

From the comparison of the content responses before and after the intervention, the nurses presented more knowledge about postpartum depression, especially in specific signs and symptoms, and improvement in the recognition and management of baby blues; they began using scales for screening and better monitoring of emotional symptoms; they began adopting more humanized practices, in addition to reporting greater safety in care and multidisciplinary integration.

**Conclusion:**

The intervention had a positive impact and expanded nurses’ knowledge, skills, and attitudes regarding postpartum depression, which contributed to more empathetic and comprehensive care. ReBEC: RBR-3hv3hhs.

## INTRODUCTION

Postpartum Depression (PPD) is a public health problem that causes profound sadness in women during the postpartum period^(1)^. Its symptoms appear within the first four weeks up to a year after delivery, and are often confused with common symptoms of Baby blues, a mild, transient condition that occurs in the first two weeks of the postpartum period due to the hormonal changes common during this period, which makes diagnosis challenging^(1,2)^.

The PPD, if not identified and treated, impacts postpartum woman’s health and well-being, leading to the development of potential behavioral, physical, and emotional problems in children^(3)^. Furthermore, the condition triggers marital and family conflicts, as it is a period of greater emotional demand for women^(4)^.

Globally, PPD affects 17.22% of women, with developing countries accounting for the highest rates of the disease^(5)^. In Brazil, the prevalence is 26%^(6)^. However, the COVID-19 pandemic has resulted in an international increase in cases of PPD and anxiety in women. Studies show prevalence rates of up to 47.2% for depression and 40.8% for anxiety, in addition to increased rates of suicidal ideation^(7,8)^.

In this context, the World Health Organization (WHO) recommends the use, by health professionals, of validated scales for tracking the disease, such as the *Edinburgh Postnatal Depression Scale* (EPDS) or the *Patient Health Questionnaire-9* (PHQ-9). Furthermore, WHO emphasizes the importance of addressing the emotional well-being of pregnant and postpartum women in all consultations^(9)^.

From this perspective, the Primary Health Care (PHC) nurse stands out among professionals in the management of PPD due to their proximity to the woman during the pregnancy-puerperal cycle, favoring the early detection of signs and symptoms indicative of the disease^(10,11)^. However, moderate knowledge on the subject and other previously unknown gaps interfere with the early identification of the disease. Among these, limitations in skills stand out, such as: early identification of PPD, feelings of insecurity; gaps in technical-scientific knowledge, lack of skills and ability in the management and effective assistance of mothers’ mental health^(12,13)^.

Therefore, there is a clear need for professional education interventions on PPD for nurses, both due to the WHO recommendation and the need for interventions on the topic and the evidence of limited training for nurses. PPD reduces the quality of life of the postpartum woman and newborn, as it interferes with the desirable standard of mental health. Furthermore, studies show that women with PPD have a 6.5 times greater risk of suicide and a prevalence of approximately 7% to 10% of suicidal ideation in the postpartum period^(14,15)^. That said, knowledge and application of correct conduct for identification and treatment improve the provision of care by nurses, to ensure continuous improvement in the development of competencies (knowledge, skills, and attitudes), and quality care and effectiveness of nursing practices.

Therefore, this study aimed to evaluate the impact of an educational intervention on nursing care for women with signs of postpartum depression for primary health care nurses.

## METHOD

### DESINGN OF STUDY

This is a quasi-experimental, before-after study^(16)^, with analysis using a qualitative method of data in the pre- and post-intervention phase and adoption of the theoretical framework in light of Philippe Perrenoud^(17)^. Furthermore, the research was registered and approved on the REBEC Platform (described in the ethical aspects phase). This design was chosen to standardize the intervention carried out. Furthermore, this was previously validated by judges to ensure rigor and methodological quality and respond to the proposed objective (described in the data collection procedures phase). Although the study had a quasi-experimental design, the evaluations before and after the intervention were qualitative in nature, as the aim was to achieve a broad and in-depth understanding of the knowledge obtained by the participants according to the theory^(17)^ adopted. Furthermore, using qualitative analysis to evaluate the effect of the intervention allowed for a holistic view of the phenomenon^(18)^. Qualitative research is recommended when the aim is to understand specific issues that require more detailed and descriptive analysis^(19)^.

Moreover, the study followed the guidelines of the *Consolidated criteria for reporting qualitative research* (COREQ).

## LOCAL

The study was carried out in the primary health care units of an inland municipality in the state of Minas Gerais, Brazil, a health care reference in the health macro-region of Triângulo do Sul, Minas Gerais, with 53 Family Health Strategy teams distributed across 29 units, with the PHC being the gateway to the Brazilian Public Health System (*SUS*) and postpartum monitoring.

## POPULATION

The total population corresponded to 96 PHC nurses who were working in the municipality during the period of this study, according to the quantity made available by the municipal health department.

## SELECTION CRITERIA

The inclusion criteria were: nurses who worked in the Primary Health Care network and who provided care to postpartum women. Exclusion criteria were: being on vacation or away from work during the research; those who failed to participate in any of the educational intervention meetings; or who, despite signing the Free and Informed Consent Form (FICF), failed to respond to any of the interviews (before or after the intervention).

## SAMPLE DEFINITION

The sample was intentionally and non-probabilistic defined. Of the 96 PHC nurses, 32 expressed interest in participating in the research; of these, 28 signed the FICF, the Free Audio Recording Consent Form, answered the semi-structured questionnaire and the guiding questions (pre-intervention interview). Of the 28 professionals, 6 were part of the pilot group (PG) and 22 were part of the intervention group (IG). Of the 22 IG participants, 1 nurse was absent due to sick leave; 6 missed at least one day of the intervention; and 1 did not participate in the post-intervention interview. Thus, the final sample consisted of 14 professionals.

## DATA COLLECTION PROCEDURES

The nurses’ contact details were requested from the Health Education Section of the Municipal Health Department of the municipality, and the invitation letter was sent via telephone contact and/or *Whatsapp* ^®^, during the months of September and October 2023. Data collection, before and after the intervention, was carried out remotely (via platform *Google Meet*) in the pre-intervention phases, between November and December 2023, and post-intervention, in April 2024, or until 30 days after the intervention.

The interviews were conducted individually and the statements were collected on a separate audio recording device, and there was no recording via the platform. Finally, to avoid the risk of possible loss of confidentiality of the collected data, the participants were coded with the letter “E”, meaning “Interviewee” (Entrevistado in Portuguese), followed by an Arabic number referring to the sequence of interviews, from E01 to E14.

For data collection, before and after, a semi-structured questionnaire prepared by the authors was used, the objective of which was to assess the knowledge, skills and attitudes of PHC nurses regarding nursing care for women with signs of PPD. The content of the data collection instruments and the educational intervention script were qualitatively validated by three judges^(20)^ selected through analysis of the Lattes resumé, all nurses, doctors, specialists in nursing, mental health and/or women’s health.

The instrument consisted of two parts, the first being based on “sociodemographic and professional” data: gender, age, time since graduation as a nurse, time working in primary care, specific courses, and/or specialization in mental health; and whether the municipal health department offered continuing education programs focused on nursing care for women with signs of postpartum depression. The second part consisted of guiding questions according to the adopted theoretical framework^(17)^: “Today, what do you know about postpartum depression?; Today, how do you seek knowledge on the topic?; Today, how do you identify a woman who presents signs of postpartum depression?; Today, what nursing care do you consider important for the care of women with signs of postpartum depression?; Today, do you notice a movement towards care for women with signs of postpartum depression at the municipal level? Or is it something new, without professional engagement? Is there an opening for exposing the topic?”

## EDUCATIONAL INTERVENTION

The planning and execution of the educational intervention were based on Philippe Perrenoud’s theory of competencies^(17)^. According to the sociologist, developing professional skills means enabling individuals to act effectively in different everyday situations, knowing how to articulate knowledge, skills and attitudes to solve different problems^(17)^.

Based on this theoretical framework, the educational intervention focused on theoretical and practical content regarding PPD: the arrival of a new baby and changes in the family context; concepts of PPD and baby blues; epidemiology; pathophysiology, signs and symptoms of the disease; serious outcomes; the impact of PPD on the health of the mother, baby, and family; public health policies focused on maternal mental health; PPD screening with validated scales; a support network for women with PPD; nursing competencies; and the flow of care for women with PPD in the municipality studied. The topics covered followed the proposed theory^(17)^ with an emphasis on concepts, practices and contexts to innovate and improve professional skills.

Furthermore, to achieve the basic theory^(17)^, situations were presented based on the daily nursing experiences and practices of PHC nurses, sharing experiences among professionals, always encouraging them to participate and jointly build the learning process.

Furthermore, the lectures and discussions were planned and implemented with the aim of encouraging the application of the acquired knowledge in several practical situations and varied contexts that could occur during nursing care for women with signs of PPD in primary health units. Also, nurses were encouraged to self-evaluate their care and what was recommended in the literature, with the aim of stimulating reflection on their skills, thus driving the continuous improvement of professional skills^(17)^.

The educational intervention was led by four facilitators, three nurses and one psychologist, professionals with master’s degrees, doctorates, experience, and specialists in the area of mental health and/or women’s health, belonging to the research group of the federal university of the municipality.

The educational intervention was carried out collectively in four meetings lasting two hours each (2 pm to 4 pm), during the month of March 2024, two in person (meeting room of the municipal health department of the municipality) and two remote ones (Platform *Google Meet*)*.* Between the first and second day of intervention there was an interval of two days, and from the second to the third day an interval of five days, and between the third and fourth an interval of two days. Presentations of slides via Power point, dynamics and computer were used. The research participants’ skills were assessed in the pre- and post-intervention phase using a qualitative analysis script (guiding questions) previously validated and evaluated in a pilot study.

## DATA ANALYSIS AND TREATMENT

Sociodemographic and professional data were stored in an *Excel*® spreadsheet, exported to the software *Statistical Package for the Social Sciences* (SPSS) (version 29), and a simple descriptive statistical analysis was performed. For the analysis of the statements, they were transcribed in full in a document from *Microsoft Word* ^®^, and analyzed qualitatively, based on thematic content analysis, by Laurence Bardin, which is based on three stages: 1- pre-analysis, 2- exploration of the material, 3- treatment of the results obtained and interpretation^(21)^. The statements were organized, coded, and categorized with the support of the ATLAS.ti software^®^, version 24.

## ETHICAL ASPECTS

The study was approved by the Ethics and Research Committee, according to CAAE no. 72614123.7.0000.5154 and opinion 6.278.334/2023, and registered on the Brazilian Clinical Trials Registry (ReBEC) platform under the registration number: RBR-3hv3hhs. Furthermore, it was carried out in accordance with Resolution 466/2012, Resolution No. 510, of April 7, 2016; and Circular Letter No. 2/2021/CONEP/SECNS/MS. Those who agreed to participate in the study were asked to sign the FICF. To ensure anonymity, participants were identified by a numeric code (ENT 01, ENT 02, ENT 03...), where ENT means “Interviewee”, respecting name confidentiality.

## RESULTS

Fourteen nurses participated in the research, 85.7% (12) of whom were female, with a predominant age range of 31 to 40 years (71.4%), and time of experience of 12 to 16 years (49.9%). Specialization course in mental health represented 21.4%. No courses held by the Health Department were reported. Chart 1 below demonstrates the sociodemographic and professional characteristics investigated.

**Chart 1:** Sociodemographic and professional characteristics of participants - Uberaba, MG, Brazil, 2025.

Variables	n (n=14)	%
**Sex**		
Female	12	85.7
Male	2	14.3
**Age range**		
Up to 30 years	1	7.2
31 - 40	10	71.4
41 - 51	3	21.4
**Time in the occupation**		
6 to 9 years	12	85.7
15 years or more	2	14.3
**Time since graduation**		
6 to 9 years	5	35.7
12 to 16 years	7	50.0
25 to 27 years	2	14.3
**Postgraduate courses in menta**l **health**		
Yes	3	21.4
No	11	78.6
**Training course by the Department of Health**		
Yes	0	0.0
No	14	100

Source: Prepared by the authors, 2025.

## PRE-INTERVENTION PHASE

In the pre-intervention phase, the prior competencies of PHC nurses in providing nursing care to women with signs of PPD were assessed in light of Philippe Perrenoud’s competency theory^(17)^. From the first assessment, prior to the intervention, but focusing on the proposed theory^(17)^, three categories emerged that demonstrated the applicability of the theory^(17)^ to the study: Nurses’ knowledge about PPD, Nurses’ skills in caring for women with signs of PPD, and Nurses’ attitudes in caring for women with signs of PPD.

## NURSES’ KNOWLEDGE OF PPD

A priori, the interviewees were able to identify some indicators of PPD based on “maternal behavior”, where the main behaviors observed stood out, such as: problems with breastfeeding, maternal inattention, perception of a weaker mother-baby bond, silence of the puerperal woman, a look of demotivation, and the presence of sunken eyes. However, other situations of the disease were less understood or emphasized by them.

*If you are experiencing symptoms of sadness, right? Depression, anxiety, if it’s... how is this bond between mother and baby?* (E04)

*If she is able to breastfeed her newborn, breastfeed her child and that, that is a factor, if she has not been able to breastfeed* (E08)

*[...] sometimes the mother, very unmotivated, looks very deeply, without reaction [...] the mother is there completely disconnected [...]* (E12)

*[...] which may have a change in her sleeping pattern, her eating habits, a change in her feelings towards the child as well, she may lose interest in activities she used to enjoy, emotional changes, irritability, become very tearful, you know? [...]* (E07)

Additionally, nurses reported limited knowledge and a lack of specific training on PPD, with their search for knowledge occurring mainly in response to an immediate need, which leads to a reactive attitude towards professional care. Other topics are also undervalued by nurses, such as “Hormonal changes”, “Baby blues”, and “Consequences of postpartum depression”, which indicates a limited focus on these topics.

*I know very little [...] when I see a patient, then I go and get it. Do you understand?* (E01)

*I’ve never provided care for a patient with postpartum depression here at the unit, right?* (E05)

*Specifically about postpartum depression, I know very little, you know? We know a little about the symptoms of depression, right?* (E11)

*To be honest, I usually look for it when I have a patient here, you know?* (E12)

*[…] because today there is that other syndrome, right, that they talk about, which I don’t know if she’ll call Blue Baby, right? which is more transient, right?* (E12)

*Sometimes the woman attempts suicide. I’ve heard some reports.* (E01)

## NURSES’ SKILLS IN NURSING CARE FOR WOMEN WITH PPD INDICATIONS

There was early identification, in the speeches, of signs and symptoms in routine care provided by nurses and other professionals, as well as the perception of the importance of multidisciplinary care, active listening, referral, and patient embracement.

*When she comes back for the heel prick test, I always talk to her and ask how she is doing. How is breastfeeding going? I keep talking. There are some that I see that are just like this.* (E03)

*With us in the monitoring, sometimes in the vaccination room [...] So, identifying when this woman comes to the unit, we end up talking, right? Know how to identify and refer, when necessary.* (E07)

*[...]we schedule an appointment with a family doctor to advise whether she needs to see a psychiatrist, whether she can continue to be followed up here with us.* (E07)

*And so, I think our work tool is this listening, this embracement, I think that is fundamental, the main thing.* (E13)

Also, the relevance of collaboration between the health team and the families of postpartum women in the perception of PPD signs and symptoms was identified. Furthermore, it is possible to perceive in the statements the importance of the active search carried out by community health agents through home visits, the need for training teams on the subject, and the lack of use of specific scales for screening PPD symptoms that could assist in care. The patient’s bond with the healthcare team was also mentioned as a strategy that enables the identification and management of PPD.

*So it’s more when the mother or family comes to us. To talk about some sign that the mother is having.* (E09)

*Because when you go to the visit, we {the team} have a different perspective, but sometimes it can go unnoticed, and then we guide the family so that if necessary, if there is any change, try to seek out the unit.* (E14)

*So, the Community Health Agent sometimes brings us something, in relation to the house, family support, so it’s more in these situations.* (E12)

*[...] provide ongoing education for community agents so they can also be alert.* (E09)

*[...] train the team [...]* (E07)

*I don’t use any scales today in the consultation, no mental health scales [...]* (E11)

*Seek to have a bond with this pregnant woman throughout the prenatal period [...]*(E05)

*I think the main care is constant monitoring* (E05)

## NURSES’ ATTITUDES TOWARDS NURSING CARE FOR WOMEN WITH INDICATIONS OF PPD

Within this main category, the following themes emerged: “Openness to training”, “Knowledge of the service flow”, “Desire to improve care” and “Team interest in the topic”. The nurses’ openness to training activities on the topic was noted.

*[...] I believe there is an opening for exposing the topic, yes, because I see that we are on the team that really likes to learn and update ourselves; however, it is a topic with very little incidence [...]* (E04)

*Look, I think there is an opening. The professionals here [...] are very open to all types of training [...]* (E06)

## POST-INTERVENTION PHASE

In the post-intervention phase, the impact of the educational intervention was evaluated using the same script as the pre-intervention phase (according to the adopted theoretical framework) on the skills of PHC nurses in providing nursing care to women with signs of postpartum depression. At this stage, the three a priori categories identified in the pre-intervention phase were identified: Nurses’ knowledge about postpartum depression; Nurses’ skills in nursing care for women with signs of postpartum depression; Nurses’ attitudes in nursing care for women with signs of postpartum depression.

## NURSES’ KNOWLEDGE ABOUT PPD

This category expresses the improvement in knowledge about PPD and the perception of the importance of using a scale for PPD screening. Furthermore, nurses were able to perceive the prevalence of PPD as a problem and differentiate it from Baby blues.

*[...] we can use the scales, there are some scales that are already validated, which we can track among our postpartum women, if any of them have an indication of postpartum depression [...]* (E03)

*[...] theoretically, I know that we have the scale that was presented to us, for postpartum depression, which is the {Edinburgh} scale [...]* (E13)

*Well, I know it {PPD} is much more present than I imagined it was.* (E02)

*[...] observe whether the symptoms will only be present in the first few days and will pass or whether they will persist, so we can characterize it as depression [...]* (E10)

*[...] I was able to differentiate baby blues from depression. Postpartum depression is a little more serious. It can last from months to 2 years after birth [...]* (E12)

After training, nurses better understood the seriousness of the problem and the importance of identifying the signs and symptoms of PPD. Furthermore, they also better understood the undervaluation and underreporting of the disease.

*[...] today I know the clinical picture, and I pay more attention after we did the training, I pay more attention to what the patient tells me [...]* (E14)

*Today, after the course, we gained extensive knowledge about the signs and symptoms [...] And now we can visualize, I can visualize the symptoms during the postpartum consultation* (E08)

*And it is a disease that often goes unnoticed, often confused with maternal overload and sometimes we don’t give it much value.* (E03)

*[...] it was a very underreported topic, we didn’t have much information, it wasn’t a concern for the nurses, the gynecologist who provides this care [...]* (E08)

It was also noted that professionals were aware of the serious outcomes of PPD, in addition to the universality of the disease.

*[...] Reaching the point of more severe depression, reaching the point of attempting self-extermination or even attacking the baby.* (E04)

*[...] more serious, which can cause greater harm to both the puerperal woman and the baby [...]* (E13)

## NURSES’ SKILLS IN NURSING CARE FOR WOMEN WITH PPD INDICATIONS

Skills such as identifying warning signs indicative of PPD, early identification of signs and symptoms, awareness of the importance of multidisciplinary care, active and qualified listening to the patient, and home visits were topics raised in the professionals’ statements.

*The woman often shows signs of sadness. Sometimes, during pregnancy, you already notice, sometimes, some signs at the end of pregnancy [...] We usually identify it right after birth, so there is the period. Women often begin to show some signs during prenatal care [...]* (E10)

*[...] how her sleep is; breastfeeding issues, if she is having difficulty, if she is having pain when breastfeeding; self-esteem issues are also very important for us to know, because when the baby is born, attention turns only to him [...] observing these issues, how her emotions are, if she is having episodes of deep sadness, crying, intrusive thoughts, that kind of thing.* (E13)

*And what is beyond our reach, we are sharing with the team.* (E07)

*[...] we also have to arrange a visit to this woman, so as not to leave her completely without a professional for many days, precisely because she will need closer monitoring [...]* (E05)

The expanded view of the puerperal woman, observing her as a whole, was also mentioned by the nurses.

*[...] we really need to broaden this perspective to see the puerperal woman, to also guide [...] when she arrives there to take the child to do the heel prick test, first I try to do... attend her too, in a comprehensive way* (E01)

*[...] after we completed the training, I pay more attention to what the patient tells me.* (E14)

Health support and guidance, as well as continuous monitoring of signs and symptoms, humanization of care and nursing support, were mentioned as essential factors in the care of postpartum women.

*[...] and we, as nurses, continue to accompany this puerperal woman through her difficulties, with guidance [...]* (E13)

*[...] we have been trying to schedule an appointment for her at least every 15 days, in addition to the weekly appointment with the psychologist.* (E14)

*[...] I think the main care would be humanization, so that we understand that that woman is going through a delicate moment in her postpartum period [...]* (E08)

## NURSES’ ATTITUDES IN NURSING CARE FOR WOMEN WITH PPD INDICATIONS

Attitudes such as the active search for knowledge and the professional’s motivation to become better were presented in the statements.

*[...] today, with the training, it was good that it directed, brought examples, new things, new bibliographies [...]* (E01)

*[...]I think it had been a long time since we had such valid training with such dedicated professionals; it was very good, very enriching* (E06)

*So, I think it’s something we have to improve, change. I think I’ve improved on that, on that issue*. (E01)

The nurses’ optimism, the discussions on the topic, and increased assurance in caring for postpartum women were also mentioned.

*We discussed it a lot and today I think I try like this, I changed my perspective.* (E01)

*So, I definitely feel more empowered to serve these women, these postpartum women.* (E07)

*[...] after the training we received, I felt much more confident about the topic [...]* (E12)

## COMPARISON OF KNOWLEDGE, SKILLS AND ATTITUDES BEFORE AND AFTER THE INTERVENTION

The comparative analysis of the pre- and post-intervention period, presented in Chart 2, shows that there was a positive impact on the knowledge, skills and attitudes of nurses regarding PPD.

**Chart 2:** Comparison of knowledge, skills, and attitudes before and after the intervention - Uberaba, MG, Brazil, 2025.

Competence	Pre-intervention	Post-intervention
**Knowledge**	Knowledge about PPD indicators based on maternal behavior. Reports of lack of information.	Expanded knowledge about PPD (signs and symptoms) and use of screening scales. Perception regarding the prevalence and clinical severity of the condition.
**Skills**	Perception of the need for early identification of signs and symptoms, importance of the multidisciplinary team and need for training.	Identifying warning signs. Perception of the importance of the multidisciplinary team, active and qualified listening and home visits. Expanded perspective, understanding of monitoring, and humanized assistance.
**Attitudes**	Demonstration of openness to training. Desire to improve care.	Understanding through the search for knowledge, motivation, increased assurance to care for the postpartum woman.

Source: Prepared by the authors, 2025.

## DISCUSSION

When relating Perrenoud’s theory^(17)^ and the research theme, it is clear that professional skills favor the resolution of various setbacks in daily activities. When carrying out their activities, nurses need to link practice to acquired knowledge, skills, and attitudes. This fact was favorable in the evaluation of this research.

The educational intervention had a positive impact on the knowledge, skills, and attitudes of PHC nurses in light of the theoretical framework^(17)^ adopted. The differences in the content analyses at the pre-intervention and post-intervention moments make the deficiencies in updating and/or knowledge on the topic before and the engagement and development of skills after the intervention clear.

After the educational intervention, the results demonstrated satisfactory improvements related to nurses’ skills in approaching women with PPD.

The training improved levels of understanding on the topic, with better understanding of the scales and how to use them for the early identification of PPD, which is in line with a study^(22)^ evaluating the effect of training on PPD for nurses to determine their value in nursing practice. The results showed that there was a significant increase in knowledge and ability to provide health education about PPD to mothers, when compared with the results analyzed in the pre- and post-test.

Likewise, the educational intervention led to an improvement in the technical skills of nurses in relation to nursing care for women with signs of PPD. With attention focused on the warning signs and symptoms of the disease, nurses mentioned deep sadness, low self-esteem, lack of interest in self-care, isolation, lack of interest in baby care and breastfeeding, lack of social and family interaction, sleep disturbances, easy crying, and intrusive thoughts. Similar symptoms were found in a review study, with the most frequently cited symptoms being: mood swings, insomnia, sadness, and uncontrollable crying, headaches and body aches, feeling of being unable to care for the baby, fear of harming the baby, guilt, anxiety, panic attacks, and even suicidal ideation^(23)^.

Active and qualified listening by nurses in mental health care from prenatal care onwards was another practice improved after continuing health education, which indicates that nursing practice is more humanized and continuous. Better collaboration was also observed between nurses and other health professionals in approaching women with signs of PPD, with comprehensive attention to their physical, emotional, and social needs. The literature states that qualified active listening can improve the effective understanding of users’ real needs and improve the effectiveness of care. Furthermore, it allows for humanized care, strengthening the bond between the professional and the user, providing a more effective and decisive reception^(24)^.

The home visit was also an enhanced activity and is seen in the literature as an opportunity for support and health education for postpartum women and their families. Studies show that women who receive home visits from health professionals are less likely to develop symptoms of postpartum depression, in addition to strengthening and encouraging better mother-baby interaction, which is essential for the well-being of both mother and baby^(25)^. Moreover, emotional and social support to the postpartum women is considered significant protective factors against PPD^(26)^. When professionals carry out home visits, there is an opportunity to screen for PPD in women who do not attend their postpartum appointment^(25,27)^.

Another highlight refers to the multidisciplinary care, which had a significant improvement in the management and flow of care for women with PPD. The work of the multidisciplinary team is crucial to providing the necessary comprehensive support that meets the physical and emotional needs of mothers, through educational and psychotherapeutic interventions, providing well-being for both mother and baby^(12,28, 29, 30, 31)^.

Another skill that showed improvement was health support and guidance, which must be clear and solve the doubts of the puerperal woman and her family. Similar findings in another study indicate that the nurses interviewed believe that health education is an important factor in preparing women for the postpartum period^(32)^.

Studies show that training nurses results in more holistic and comprehensive patient care, reducing barriers to identifying and managing the disease by these professionals^(33)^. In addition, women’s mental health-focused training allows for greater assurance and empowerment in the practices carried out by nurses, which makes the implementation of screening strategies and nursing interventions more effective^(12)^.

Furthermore, a greater motivation to seek continuous knowledge on the topic was observed, as well as a more proactive and optimistic attitude towards managing the disease, after the educational intervention.

Finally, if left untreated, PPD clearly impacts maternal mental health in the postpartum period, with negative repercussions for both the woman and the family^(34)^. Therefore, the importance of addressing this condition, which is a public health problem, is emphasized. Consequently, it is essential that municipal health departments develop a training program for PHC professionals, to support improvements in the care provided by the *SUS*, in accordance with the recommendations of the National Policy for Continuing Education in Health (Ordinance No. 198/GM of February 13, 2004)^(35)^.

The study’s limitations include low adherence among nurses, as the virtual environment remains an obstacle due to unstable internet connections, and the fact that the research was conducted only with nurses, without involving other professionals who assist postpartum women in PHC. However, the professionals who participated in the research were involved, interested in the topic and adopted intervention, which contributes to the quality of care in PHC.

## CONCLUSION

In conclusion, the objective proposed by the study was achieved. The educational intervention on PPD had a positive impact, as it expanded knowledge, skills, practices and, above all, improved nurses’ attitudes, enabling more empathetic, humanized and technically based care.

Considering the importance of the topic, we suggest that further studies are carried out and other strategies are developed to engage health professionals and identify the best interventions and ways to promote postpartum women’s mental health and well-being.

## Data Availability

https://doi.org/10.48331/SCIELODATA.LQRF9N
