# ERRATUM: Impact of an educational intervention on postpartum depression for primary care nurses: a quasi-experimental study

**DOI:** 10.1590/1980-220X-REEUSP-2025-0032eren

**Published:** 2025-12-01

In the article “Impact of an educational intervention on postpartum depression for primary care nurses: a quasi-experimental study”, with DOI code number: https://doi.org/10.1590/1980-220X-REEUSP-2025-0032en, published in the journal Revista da Escola de Enfermagem da USP, 59:e20250032, 2025, in the page 1:

**Where it was written:**

**Corresponding author:**

Débora Alves da Silva

Rua Lourival Pereira Gomes, 570, Parque das Laranjeiras

04764-070 – Uberaba, MG, Brazil

dalvesenf@gmail.com

**Should read:**

**Corresponding author:**

Débora Alves da Silva

Av. Getúlio Guaritá, 107, Nossa Sra. da Abadia

38025-440 – Uberaba, MG, Brazil

dalvesenf@gmail.com

Prof. Dr. Maria Amélia de Campos Oliveira

*Editor-in-chief*

