# Supplementary material for: Impact of an educational intervention on postpartum depression for primary care nurses: a quasi-experimental study
Source: Rev Esc Enferm USP. 2025 Sep 8;59:e20250032. doi: 10.1590/1980-220X-REEUSP-2025-0032en (PMC12416864; doi:10.1590/1980-220X-REEUSP-2025-0032en)
Supplement: Translation

# Translation: Impacto de uma intervenção educativa sobre depressão pós-parto para enfermeiros da atenção primária: estudo quase-experimental

**DOI:** 10.1590/1980-220X-REEUSP-2025-0032pt

**Authors:** Débora Alves da Silva, Marli Aparecida Reis Coimbra, Lucas Carvalho Santana, Maria Aline Leocádio, Fernanda Bonato Zuffi, Lúcia Aparecida Ferreira

**Affiliations:** 1 Universidade Federal do Triângulo Mineiro, Uberaba, MG, Brasil.; 2 Universidade Federal do Triângulo Mineiro, Hospital de Clínicas, Uberaba, MG, Brazil.

**Keywords:** Enfermeiras e Enfermeiros, Depressão Pós-Parto, Educação Continuada, Atenção Primária à Saúde, Ensino

## Abstract

**Objetivo:**

Avaliar o impacto de uma intervenção educativa sobre a assistência de enfermagem à mulher com indicativo de depressão pós-parto para enfermeiros da atenção primária à saúde.

**Método:**

Estudo quase-experimental, do tipo antes e depois, realizado com 14 enfermeiros da atenção primária à saúde de um município que participaram de uma intervenção educativa sobre a assistência de enfermagem à mulher com indicativo de depressão pós-parto. Realizada análise qualitativa dos dados antes e depois da intervenção, por meio de análise de conteúdo temática de Bardin.

**Resultados:**

Da comparação das respostas dos conteúdos antes e depois da intervenção, os enfermeiros apresentaram mais conhecimento sobre a depressão pós-parto, especialmente em sinais e sintomas específicos, e melhora no reconhecimento e manejo do *baby blues*; passaram a usar escalas para o rastreamento, monitorar melhor os sintomas emocionais; começaram a adotar práticas mais humanizadas, além de relatarem maior segurança no atendimento e integração multiprofissional.

**Conclusão:**

A intervenção impactou positivamente e ampliou o conhecimento, habilidades e atitudes dos enfermeiros sobre a depressão pós-parto, o que contribuiu para uma assistência mais empática e integral. ReBEC: RBR-3hv3hhs.

## INTRODUÇÃO

A Depressão Pós-Parto (DPP) é um problema de saúde pública que causa um quadro de profunda tristeza às mulheres no puerpério^(1)^. Seus sintomas aparecem entre as primeiras quatro semanas até um ano após o parto, e muitas vezes são confundidos com sintomas comuns do *Baby blues*, condição transitória e branda que ocorre nas duas primeiras semanas de puerpério devido às alterações hormonais comuns neste período, o que torna o diagnóstico desafiador^(1,2)^.

A DPP, caso não seja identificada e tratada, impacta a saúde e bem-estar da puérpera, além de levar ao desenvolvimento de potenciais problemas comportamentais, físicos, e emocionais nas crianças^(3)^. Além disso, a condição desencadeia conflitos conjugais e familiares, por ser um período de maior demanda emocional pela mulher^(4)^.

Globalmente, a DPP atinge 17,22% das mulheres, sendo os países em desenvolvimento responsáveis pelas maiores taxas da doença^(5)^. No Brasil, a prevalência é de 26%^(6)^. Todavia, a pandemia de COVID-19 refletiu internacionalmente no aumento dos casos de DPP e ansiedade em mulheres. Os estudos apresentam prevalências de até 47,2% de depressão e 40,8% de ansiedade, além do aumento nos índices de ideação suicida^(7,8)^.

Diante desse contexto, a Organização Mundial de Saúde (OMS) recomenda o uso de escalas validadas para o rastreamento da doença pelos profissionais de saúde, como a *Edinburgh Postnatal Depression Scale* (EPDS) ou a *Patient Health Questionnaire-9* (PHQ-9). Ainda, a OMS enfatiza a importância de abordar o bem-estar emocional de gestantes e puérperas em todas as consultas^(9)^.

Nessa perspectiva, o enfermeiro da Atenção Primária à Saúde (APS) destaca-se entre os profissionais no manejo da DPP devido a sua proximidade com a mulher durante o ciclo gravídico-puerperal, favorecendo a detecção precoce de sinais e sintomas indicativos da doença^(10,11)^. Entretanto, o conhecimento moderado sobre a temática e outras lacunas até então desconhecidas interferem na identificação precoce da doença. Entre essas, destacam-se limitações em competências, como: identificação precoce da DPP, sentimento de insegurança; falta de habilidades e lacunas no conhecimento técnico-científico e no gerenciamento e assistência eficaz à saúde mental das mães^(12,13)^.

Portanto, percebe-se a necessidade de intervenções de educação profissional sobre a DPP para os enfermeiros, tanto pela recomendação da OMS, quanto pela necessidade de intervenções sobre o tema e pelas evidências de formação limitada de enfermeiros. A DPP reduz a qualidade de vida da puérpera e do recém-nascido, pois interfere no padrão de saúde mental desejável. Ademais, estudos mostram que mulheres com DPP apresentam um risco 6,5 vezes maior para suicídio e uma prevalência de aproximadamente 7% a 10% de ideação suicida no puerpério^(14,15)^. Isto posto, o conhecimento e aplicação de condutas corretas para identificação e tratamento aprimoram a prestação de cuidados pelos enfermeiros, a fim de garantir a melhoria contínua no desenvolvimento de competências (conhecimento, habilidades e atitudes) e a assistência de qualidade e eficácia das práticas de enfermagem.

Destarte, este estudo teve como objetivo avaliar o impacto de uma intervenção educativa sobre a assistência de enfermagem à mulher com indicativo de depressão pós-parto para enfermeiros da atenção primária à saúde.

## MÉTODO

### TIPO DE ESTUDO

Trata-se de um estudo quase-experimental^(16)^, do tipo antes e depois, com análise por meio de método qualitativo dos dados na fase pré e pós-intervenção e adoção do referencial teórico à luz de Philippe Perrenoud^(17)^. Além disso, a pesquisa obteve registro e aprovação na Plataforma REBEC (descrita na fase aspectos éticos). Escolheu-se esse delineamento a fim de padronizar a intervenção realizada. Além disso, esta foi previamente validada por juízes para garantir o rigor e qualidade metodológica e responder ao objetivo proposto (descrito na fase procedimentos para coleta de dados). Embora o estudo seja de delineamento quase-experimental, as avaliações antes e depois da intervenção foram de cunho qualitativo, pois buscou-se a compreensão ampla e profunda do conhecimento obtido pelos participantes segundo a teoria^(17)^ adotada. Além disso, utilizar a análise qualitativa para avaliar o efeito da intervenção permitiu uma visão holística do fenômeno^(18)^. Pesquisas qualitativas são indicadas quando se objetiva compreender questões específicas que exigem análise mais detalhada e descritiva^(19)^.

Outrossim, o estudo foi norteado pelas diretrizes do *Consolidated criteria for reporting qualitative research* (COREQ).

## LOCAL

O estudo foi realizado nas unidades básicas de saúde de um município do interior de Minas Gerais, Brasil, referência de saúde da macrorregião de saúde do Triângulo do Sul, Minas Gerais, com 53 equipes da Estratégia Saúde da Família distribuídas em 29 unidades, sendo a APS a porta de entrada para o Sistema Único de Saúde (SUS) e acompanhamento puerperal.

## POPULAÇÃO

A população total correspondeu a 96 enfermeiros da APS que estavam trabalhando no município no período da realização deste estudo, conforme quantitativo disponibilizado pela secretaria municipal de saúde.

## CRITÉRIOS DE SELEÇÃO

Considerou-se como critério de inclusão: enfermeiros que atuavam na rede de Atenção Primária à Saúde e que realizavam atendimento às puérperas. Como critérios de exclusão: estar em período de férias ou afastado das suas atividades laborais, durante a realização da pesquisa; aqueles que deixaram de participar de algum dos encontros da intervenção educativa; ou que apesar de assinar o Termo de Consentimento Livre e Esclarecido (TCLE), deixaram de responder alguma das entrevistas (antes ou após a intervenção).

## DEFINIÇÃO DA AMOSTRA

A amostra foi definida de modo intencional e não probabilístico. Dos 96 enfermeiros da APS, 32 manifestaram interesse em participar da pesquisa; desses, 28 assinaram o TCLE, Termo de Autorização de uso de Voz e Som (TAVS), responderam ao questionário semiestruturado e às perguntas norteadoras (entrevista pré-intervenção). Dos 28 profissionais, 06 integraram o grupo piloto (GP) e 22 o grupo de intervenção (GI). Dos 22 participantes do GI, 01 enfermeiro ausentou-se devido a licença saúde; 06 faltaram pelo menos um dia da intervenção; e 01 não participou da entrevista pós-intervenção. Assim, a amostra final foi de 14 profissionais.

## PROCEDIMENTOS PARA COLETA DE DADOS

Os contatos dos enfermeiros foram solicitados à Seção de Educação em Saúde da Secretaria Municipal de Saúde do município, e a carta convite enviada via contato telefônico e/ou *Whatsapp* ^®^, durante os meses de setembro e outubro de 2023. A coleta de dados, antes e depois da intervenção, foi realizada remotamente (via plataforma *Google meet*) nas fases pré-intervenção, entre novembro e dezembro de 2023, e pós-intervenção, em abril de 2024, ou até que se completassem 30 dias após a intervenção.

As entrevistas foram realizadas individualmente e os depoimentos foram coletados em dispositivo de gravação de áudio avulso, e não houve gravação pela plataforma. Por fim, para evitar o risco de possível perda da confidencialidade dos dados coletados, os participantes foram codificados com a letra “E”, significando “Entrevistado”, seguida de um número arábico referente à sequência das entrevistas, de E01 a E14.

Para coleta de dados, antes e após, utilizou-se questionário semiestruturado elaborado pelos autores, cujo objetivo foi avaliar o conhecimento, habilidades e atitudes dos enfermeiros da APS quanto à assistência de Enfermagem à mulher com indicativo de DPP. O conteúdo dos instrumentos de coleta de dados e o roteiro da intervenção educativa foram validados, qualitativamente, por três juízes^(20)^ selecionados por meio de análise do currículo Lattes, todos enfermeiros, doutores, especialistas em enfermagem, saúde mental e/ou saúde da mulher.

O instrumento foi composto por duas partes, sendo a primeira baseada nos dados “sociodemográficos e profissionais”: gênero, idade, tempo de formação como enfermeiro, tempo de atuação na atenção primária, cursos e/ou especialização específicos em saúde mental; e se a secretaria municipal de saúde oferecia educação permanente voltada ao atendimento do enfermeiro à mulher com indicativo de depressão pós-parto. A segunda parte foi composta pelas perguntas norteadoras segundo o referencial teórico adotado^(17)^: “Hoje, o que você sabe sobre a depressão pós-parto?; Hoje, como você busca o conhecimento sobre o tema?; Hoje, como você identifica uma mulher que apresenta indicativo de depressão pós-parto?; Hoje, quais os cuidados de enfermagem que você considera importantes para a assistência de enfermagem à mulher com indicativo de depressão pós-parto?; Hoje, você percebe um movimento para a atenção à mulher com indicativo de depressão pós-parto no âmbito municipal? Ou é algo novo, sem engajamento dos profissionais? Há abertura para exposição do tema?”.

## INTERVENÇÃO EDUCATIVA

O planejamento e execução da intervenção educativa foram fundamentados na teoria das competências de Philippe Perrenoud^(17)^. Segundo o sociólogo, desenvolver competências profissionais é capacitar os indivíduos a agirem eficazmente em diferentes situações do cotidiano, sabendo articular o conhecimento, habilidade e atitudes na solução de problemas diversos^(17)^.

Fundamentada nesse referencial teórico, a intervenção educativa teve como conteúdo teórico-prático sobre a DPP: a chegada de um novo bebê e as mudanças no contexto familiar; conceitos de DPP e Baby blues; epidemiologia; fisiopatologia, sinais e sintomas da doença; desfechos graves; impacto da DPP na saúde da mãe, do bebê e no núcleo familiar; políticas públicas de saúde voltadas à saúde mental materna; rastreamento da DPP com escalas validadas; rede de apoio à mulher com DPP; competências do enfermeiro; e fluxo de atendimento à mulher com DPP no município estudado. Os temas abordados seguiram a teoria proposta^(17)^ com ênfase nos conceitos, práticas e contextos para inovar e aprimorar as competências profissionais.

Ademais, para se alcançar a teoria de base^(17)^, foram apresentadas situações baseadas nas experiências e práticas de enfermagem diárias dos enfermeiros da APS, compartilhamento de vivências entre os profissionais, sempre os estimulando a participarem e a construírem conjuntamente o processo de aprendizagem.

Além disso, as palestras e discussões foram planejadas e implementadas com o objetivo de estimular a aplicação do conhecimento adquirido nas várias situações práticas e contextos variados que poderiam ocorrer durante o atendimento de enfermagem à mulher com indicativos de DPP nas unidades básicas de saúde. Também, estimulou-se a autoavaliação do enfermeiro quanto ao seu atendimento realizado e ao que era preconizado na literatura, com o intuito de estimular a reflexão de suas competências, assim impulsionando a melhoria contínua das competências profissionais^(17)^.

A intervenção educativa foi conduzida por quatro facilitadoras, sendo três enfermeiras e uma psicóloga, profissionais com mestrado, doutorado, experiência e especialistas na área de saúde mental e/ou saúde da mulher, pertencentes ao grupo de pesquisa da universidade federal do município.

A intervenção educativa foi realizada, coletivamente, em quatro encontros com duração de duas horas cada um (14h às 16h), durante o mês de março de 2024, sendo dois presenciais (sala de reuniões da secretaria municipal de saúde do município) e dois remotos *(*Plataforma *Google meet),* sendo que entre o primeiro e o segundo dia de intervenção houve um intervalo de dois dias, e do segundo para o terceiro dia um intervalo de cinco dias, e entre o terceiro e quarto um intervalo de dois dias. Utilizaram-se apresentações de *slides* via *Power Point,* dinâmica e computador. As competências dos participantes da pesquisa foram avaliadas na fase pré e pós-intervenção pelo roteiro de análise qualitativa (perguntas norteadoras) previamente validado e avaliado em estudo piloto.

### ANÁLISE E TRATAMENTO DOS DADOS

Os dados sociodemográficos e profissionais foram armazenados em planilha do *Excel*®, exportados para o *Statistical Package for the Social Sciences* (SPSS) (versão 29), e realizada análise estatística descritiva simples. Para a análise dos depoimentos, estes foram transcritos na íntegra em documento do *Microsoft Word* ^®^, e analisados qualitativamente, fundamentados na Análise de conteúdo temática, de Laurence Bardin, que se baseia em três etapas: 1- pré-análise, 2- exploração do material, 3- tratamento dos resultados obtidos e interpretação^(21)^. Os depoimentos foram organizados, codificados e categorizados com o suporte do software ATLAS.ti^®^, versão 24.

## ASPECTOS ÉTICOS

O estudo teve aprovação do Comitê de Ética e Pesquisa, conforme CAAE nº 72614123.7.0000.5154 e parecer 6.278.334/2023, e registro na plataforma de Registro Brasileiro de Ensaios Clínicos (ReBEC) sob o número de registro: RBR-3hv3hhs. Também, foi realizado em conformidade à Resolução 466/2012, Resolução nº 510, de 07 de abril de 2016; e Ofício Circular Nº 2/2021/CONEP/SECNS/MS. Além disso, àqueles que aceitaram participar do estudo, foi solicitada anuência mediante assinatura do TCLE, e para assegurar o anonimato, os participantes foram identificados por meio de um código numérico (ENT 01, ENT 02, ENT 03...), onde ENT significa “Entrevistado”, respeitando-se o sigilo dos nomes.

## RESULTADOS

Catorze enfermeiros participaram da pesquisa, sendo 85,7% (12) do sexo feminino, com faixa etária predominante de 31 a 40 anos (71,4%), tempo de atuação de 12 a 16 anos (49,9%). Curso de especialização em saúde mental representou 21,4%. Não foram relatados cursos realizados pela Secretaria de Saúde. O Quadro 1 abaixo demonstra as características sociodemográficas e profissionais investigadas.

**Quadro 1:** Características sociodemográficas e profissionais dos participantes - Uberaba, MG, Brasil, 2025.

Variáveis	n (n = 14)	%
**Sexo**		
Feminino	12	85,7
Masculino	2	14,3
**Faixa etária**		
Até 30 anos	1	7,2
31 - 40	10	71,4
41 - 51	3	21,4
**Tempo de atuação**		
6 a 9 anos	12	85,7
15 anos ou +	2	14,3
**Tempo de formação**		
6 a 9 anos	5	35,7
12 a 16 anos	7	50,0
25 a 27 anos	2	14,3
**Cursos de pós-graduação em saúde mental**		
Sim	3	21,4
Não	11	78,6
**Curso de capacitação pela Secretaria de Saúde**		
Sim	0	0,0
Não	14	100

Fonte: Elaborado pelos autores, 2025.

## FASE PRÉ-INTERVENÇÃO

Na fase pré-intervenção, foram avaliadas as competências prévias dos enfermeiros da APS na assistência de enfermagem à mulher com indicativos de DPP à luz da teoria das competências de Philippe Perrenoud^(17)^. Da primeira avaliação, prévia à intervenção, porém com foco na teoria proposta^(17)^, emergiram três categorias que demonstraram a aplicabilidade da teoria^(17)^ ao estudo: Conhecimento dos Enfermeiros sobre a DPP, Habilidades dos Enfermeiros na assistência à mulher com indicativos de DPP, e Atitudes dos Enfermeiros na assistência à mulher com indicativos de DPP.

## CONHECIMENTO DOS ENFERMEIROS SOBRE A DPP

A priori, os entrevistados conseguiam identificar alguns indicativos de DPP baseados no “comportamento materno”, onde se destacavam como os principais comportamentos observados: problemas com a amamentação, desatenção da mãe, percepção de menor vínculo mãe-bebê, silêncio da puérpera, olhar de desmotivação, e presença de olho fundo. Entretanto, outras situações da doença eram menos compreendidas ou enfatizadas por eles.

*Se está com sintomas de tristeza, né? De depressão, ansiedade, se tá… como está esse vínculo entre mãe e bebê?* (E04)

*Se ela tá conseguindo amamentar o seu recém-nascido, amamentar a sua criança e isso, isso é um fator, se ela não tiver conseguido amamentar* (E08)

*[...] às vezes a mãe, muito desmotivada, olho muito fundo, sem reação [...] mãe tá ali totalmente desligada [...]* (E12)

*[...] que pode ter alteração do padrão de sono, da alimentação dela, uma alteração assim em relação aos sentimentos com a criança também, ela pode ter perda de interesse pelas atividades que ela gostava de fazer, alteração emocional, irritabilidade, ficar muito chorosa, né? [...]* (E07)

Outrossim, os enfermeiros relataram conhecimento limitado e carência de formação específica sobre a DPP, além de a busca pelo conhecimento ocorrer principalmente diante de uma necessidade imediata, o que traz uma postura reativa ao atendimento do profissional. Notam-se ainda outros temas subvalorizados pelos enfermeiros, como “Alterações hormonais”, “Baby blues”, e “Consequências da depressão pós-parto”, o que indica um enfoque limitado nesses tópicos.

*Eu sei muito pouco [...] quando eu atendo alguma paciente, aí eu vou buscar. Entendeu?* (E01)

*Eu nunca cuidei de uma paciente com depressão pós-parto aqui na unidade, né?* (E05)

*Especificamente sobre a depressão pós-parto eu sei muito pouco, né? A gente sabe um pouco dos sintomas depressivos, né?* (E11)

*Pra falar a verdade mesmo, geralmente eu busco quando eu tenho alguma paciente aqui, né?* (E12)

*[…] porque hoje tem aquela outra síndrome, né, que eles falam, que eu não sei se ela vai chamar Blue Baby, né? que é mais o passageiro, né?* (E12)

*Às vezes a mulher tenta suicídio. Já ouvi alguns relatos.* (E01)

## HABILIDADES DOS ENFERMEIROS NA ASSISTÊNCIA DE ENFERMAGEM À MULHER COM INDICATIVO DE DPP

Houve a identificação precoce, nas falas, de sinais e sintomas nos atendimentos de rotina do enfermeiro e outros profissionais, bem como a percepção da importância do atendimento multiprofissional, da escuta ativa, do encaminhamento e acolhimento da paciente.

*Quando ela volta lá no teste do pezinho, eu sempre converso com ela e pergunto como que tá? Como é que tá a amamentação? Vou conversando. Tem umas que eu vejo que tá bem assim.* (E03)

*Com a gente no acompanhamento, às vezes na sala de vacina [...] Então, identificar quando essa mulher vem até a unidade, a gente acaba conversando, né? Saber identificar e encaminhar, quando necessário.* (E07)

*[...]a gente marca uma consulta com um médico da família para estar orientando se precisa passar por psiquiatra, se ela consegue continuar acompanhando aqui com a gente.* (E07)

*E assim, eu acho que a nossa ferramenta de trabalho é essa escuta, é esse acolhimento, eu acho que é o fundamental, o principal.* (E13)

Também, identificou-se a relevância da colaboração entre a equipe de saúde e os familiares das puérperas na percepção dos sinais e sintomas da DPP. Além disso, é possível perceber nas falas a importância da busca ativa realizada pelos agentes comunitários de saúde a partir das visitas domiciliares, a necessidade de capacitação das equipes sobre a temática, e a falta da utilização de escalas específicas para o rastreio de sintomas de DPP que poderiam auxiliar nos atendimentos. O vínculo da paciente com a equipe de saúde também foi mencionado como uma estratégia que possibilita a identificação e o manejo da DPP.

*Então é mais quando a mãe ou a família nos procura. Para falar de algum sinal que a mãe está tendo.* (E09)

*Porque na hora que você vai fazer a visita, a gente {equipe} tem um olhar diferenciado, mas às vezes pode passar batido, e aí a gente orienta a família no sentido de se precisar, se apresentar alguma alteração, tentar ir buscando a unidade.* (E14)

*Então, o Agente Comunitário de Saúde, às vezes traz alguma coisa pra gente, em relação à casa, apoio familiar, então é mais nessas situações.* (E12)

*[...] fazer uma educação continuada com os agentes comunitários pra eles também ficarem atentos.* (E09)

*[...] capacitar a equipe [...]* (E07)

*Eu não utilizo nenhuma escala hoje na consulta, nenhuma escala de saúde mental não [...]* (E11)

*Buscar ter um vínculo com essa gestante durante todo o pré-natal [...]* (E05)

*Acho que o principal cuidado é o acompanhamento constante* (E05)

## ATITUDES DOS ENFERMEIROS PARA A ASSISTÊNCIA DE ENFERMAGEM À MULHER COM INDICATIVO DE DPP

Dentro desta categoria principal surgiram os temas: “Abertura à capacitação”, “Conhecer o fluxo de atendimento”, “Desejo por melhorar a assistência” e “Interesse da equipe sobre o tema”. Percebeu-se a abertura dos enfermeiros para atividades de capacitação sobre a temática.

*[...] acredito que há uma abertura para exposição do tema sim, porque eu vejo que nós estamos na equipe que gosta bastante de aprender e se atualizar; porém, é um tema com pouquíssima incidência [...]* (E04)

*Olha, eu acho que abertura tem. Os profissionais aqui [...] são bem abertos a todos os tipos de capacitações [...]* (E06)

## FASE PÓS-INTERVENÇÃO

Na fase pós-intervenção, foi avaliado o impacto da intervenção educativa com o mesmo roteiro da fase pré-intervenção (segundo o referencial teórico adotado) nas competências dos enfermeiros da APS na assistência de enfermagem à mulher com indicativo de depressão pós-parto. Nessa etapa identificaram-se as três categorias apriorísticas identificadas na fase de pré-intervenção: Conhecimentos dos Enfermeiros sobre a Depressão pós-parto; Habilidades dos Enfermeiros na assistência de enfermagem à mulher com indicativo de Depressão pós-parto; Atitudes dos Enfermeiros na assistência de enfermagem à mulher com indicativo de Depressão pós-parto.

## CONHECIMENTOS DOS ENFERMEIROS SOBRE A DPP

Essa categoria expressa a melhora do conhecimento sobre DPP e a percepção da importância do uso de escala para o rastreio de DPP. Além disso, os enfermeiros conseguiram perceber a prevalência da DPP como um problema e diferenciá-la do *Baby blues*.

*[...] a gente pode utilizar as escalas, existem algumas escalas que já são validadas, que a gente pode rastrear entre as nossas puérperas, se alguma tem um indicativo de depressão pós-parto [...]* (E03)

*[...] teoricamente, eu sei que a gente tem a escala que foi apresentada para a gente, de depressão pós-parto, que é a de {Edimburgo} [...]* (E13)

*Bom, eu sei que ela {DPP} é muito mais presente do que eu imaginei que era.* (E02)

*[...]observar se os sintomas serão só nos primeiros dias e vai passar ou se vai persistir, para a gente caracterizar como uma depressão [...]* (E10)

*[...] consegui diferenciar o baby blues da depressão. A depressão pós-parto é um pouco mais grave. Ela pode durar de meses até 2 anos após o parto [...]* (E12)

Após a capacitação, os enfermeiros compreenderam melhor a seriedade do problema e a importância de identificar os sinais e sintomas da DPP. Outrossim, eles também perceberam melhor a subvalorização e subnotificação da doença.

*[...] hoje eu sei do quadro clínico, e eu tenho mais atenção depois que a gente fez a capacitação, eu presto mais atenção no que a paciente me relata [...]* (E14)

*Hoje, após o curso, a gente teve um amplo conhecimento a respeito dos sinais e sintomas [...] E agora a gente consegue visualizar, eu consigo visualizar os sintomas durante a consulta puerperal* (E08)

*E é uma doença que muitas das vezes passa despercebida, muito confundida com a sobrecarga materna e às vezes a gente não dá tanto valor.* (E03)

*[...] era um tema muito subnotificado, a gente não tinha muitas informações, não era uma preocupação dos enfermeiros, do ginecologista que faz esse atendimento [...]* (E08)

Percebeu-se também a conscientização dos profissionais sobre os desfechos graves da DPP, além da universalidade da doença.

*[...] Chegar ao ponto de uma depressão mais grave, chegar ao ponto de uma tentativa de autoextermínio ou até mesmo de agressão ao bebê.* (E04)

*[...] mais agravados, que podem trazer prejuízos maiores tanto para a puérpera quanto para o bebê [...]* (E13)

## HABILIDADES DOS ENFERMEIROS NA ASSISTÊNCIA DE ENFERMAGEM À MULHER COM INDICATIVO DE DPP

Habilidades como a identificação de sinais de alerta indicativos de DPP, a identificação precoce dos sinais e sintomas, ter consciência da importância do atendimento multiprofissional, da escuta ativa e qualificada do paciente, e a visita domiciliar foram temas levantados nos depoimentos dos profissionais.

*A mulher costuma apresentar sinais de tristeza. Às vezes, durante a gravidez, você já percebe, às vezes, alguns sinais no final da gravidez [...] A gente costuma identificar ela logo após o parto, então tem o período. A mulher muitas das vezes começa a dar algum sinal durante o pré-natal [...]* (E10)

*[...] como que está o sono dela; questão da amamentação, se tá tendo dificuldade, se tá tendo dor para amamentar; questão de autoestima também é muito importante a gente saber, porque quando o bebê nasce, as atenções voltam somente para ele [...] observando essas questões, como estão as emoções dela, se ela está tendo episódios de tristeza profunda, de choro, de pensamentos intrusivos, isso.* (E13)

*E o que não está ao nosso alcance, a gente está dividindo com a equipe.* (E07)

*[...] também já organizar para fazer uma visita para essa mulher, para não deixar ela totalmente sem profissional por muitos dias, justamente porque ela vai precisar de acompanhamento mais de perto [...]* (E05)

O olhar ampliado à puérpera, observando-a integralmente, também foi mencionado pelos enfermeiros.

*[...] a gente precisa realmente ampliar esse olhar para ver a puérpera, para conduzir também [...] quando ela chega lá para levar a criança para fazer o teste do pezinho, primeiro eu procuro fazer... atender ela mesmo, de forma integral* (E01)

*[...] depois que a gente fez a capacitação, eu presto mais atenção no que a paciente me relata.* (E14)

O apoio e a orientação de saúde, como também o monitoramento contínuo dos sinais e sintomas, a humanização da assistência e o suporte da enfermagem, foram mencionados como fatores essenciais no cuidado à puérpera.

*[...] e nós, como enfermeiros, continuar acompanhando essa puérpera nas dificuldades dela, com orientações [...]* (E13)

*[...] a gente tem procurado agendar consulta para ela pelo menos de 15 em 15 dias, fora o atendimento semanal com a psicóloga.* (E14)

*[...] eu acho que o principal cuidado seria a humanização, para a gente entender que aquela mulher, ela está passando por um momento delicado do pós-parto dela [...]* (E08)

## ATITUDES DOS ENFERMEIROS NA ASSISTÊNCIA DE ENFERMAGEM À MULHER COM INDICATIVO DE DPP

Atitudes como a busca ativa pelo conhecimento e a motivação do profissional para se tornar melhor foram apresentadas nos depoimentos.

*[...] hoje, com a capacitação, foi bom que direcionou, trouxe exemplos, coisas novas, novas bibliografias [...]* (E01)

*[...]acho que tinha bastante tempo que a gente não tinha uma capacitação tão válida com profissionais tão empenhados; foi muito boa, muito rica* (E06)

*Então, acho que é uma coisa que a gente tem que ir melhorando, mudando. Eu acho que eu melhorei nisso, nessa questão*. (E01)

O otimismo dos enfermeiros, as discussões sobre a temática e o aumento da segurança para o atendimento das puérperas, também foram mencionados.

*A gente discutiu bastante e hoje eu acho que eu tento assim, eu mudei o meu olhar.* (E01)

*Então, eu me sinto mais empoderada para atender essas mulheres, com certeza, essas puérperas.* (E07)

*[...] após o treinamento que a gente recebeu, eu fiquei bem mais segura em relação ao tema [...]* (E12)

## COMPARATIVO DOS CONHECIMENTOS, HABILIDADES E ATITUDES NA PRÉ E PÓS-INTERVENÇÃO

A análise comparativa do momento pré e pós-intervenção, apresentada no Quadro 2, evidencia que houve impacto positivo no conhecimento, habilidades e atitudes dos enfermeiros para a atuação frente à DPP.

**Quadro 2:** Comparativo do conhecimento, habilidades e atitudes na pré e pós-intervenção - Uberaba, MG, Brasil, 2025.

Competência	Pré-intervenção	Pós-intervenção
**Conhecimento**	Conhecimento sobre indicativo de DPP baseado no comportamento materno. Relatos de carência de informação.	Conhecimento ampliado sobre DPP (sinais e sintomas) e uso de escalas de rastreamento. Percepção quanto à prevalência e à gravidade clínica da condição.
**Habilidades**	Percepção da necessidade de identificação precoce de sinais e sintomas, importância da equipe multiprofissional e necessidade de treinamento.	Identificação de sinais de alerta. Percepção da importância da equipe multiprofissional, da escuta ativa e qualificada e da visita domiciliar. Olhar ampliado, entendimento do monitoramento e assistência humanizada.
**Atitudes**	Demonstração de abertura para a capacitação. Desejo de melhorar a assistência.	Entendimento pela busca de conhecimento, motivação, aumento da segurança para atender a puérpera.

Fonte: Elaborado pelos autores, 2025.

## DISCUSSÃO

Ao relacionar a teoria de Perrenoud^(17)^ e a temática da pesquisa, tem-se que as competências profissionais favorecem a resolução de diversos percalços nas atividades cotidianas. Durante a execução de suas atividades os enfermeiros precisam articular a prática ao conhecimento, às habilidades e às atitudes adquiridas. Fato que foi favorável na avaliação desta pesquisa.

A intervenção educativa apresentou impacto positivo no conhecimento, habilidades e atitudes dos enfermeiros da APS à luz do referencial teórico^(17)^ adotado. As diferenças das análises dos conteúdos nos momentos pré-intervenção e pós-intervenção deixam claras as deficiências de atualização e/ou conhecimento sobre o tema antes e o engajamento e desenvolvimento de habilidades depois da intervenção.

Após a intervenção educativa, os resultados demonstraram satisfatoriamente melhorias relacionadas às competências dos enfermeiros na abordagem às mulheres com DPP.

A capacitação melhorou os níveis de compreensão sobre o tema, com melhor entendimento sobre as escalas e como utilizá-las para a identificação precoce da DPP. O que vai ao encontro de um estudo^(22)^ que avaliou o efeito de uma capacitação sobre DPP para enfermeiros a fim de determinar o seu valor na prática de enfermagem. Os resultados mostraram que houve aumento significativo do conhecimento e da capacidade de fornecer educação em saúde sobre DPP às mães, quando comparado com os resultados analisados no pré e pós-teste.

Além disso, a intervenção educativa propiciou uma melhora nas habilidades técnicas dos enfermeiros em relação à assistência de enfermagem à mulher com indicativos de DPP. Sendo a atenção direcionada aos sinais e sintomas de alerta da doença, os enfermeiros mencionaram tristeza profunda, baixa autoestima, desinteresse pelo autocuidado, isolamento, desinteresse pelos cuidados do bebê e com a amamentação, falta de interação social e familiar, distúrbios do sono, choro fácil, e pensamentos intrusivos. Sintoma semelhantes foram encontrados em um estudo de revisão, sendo os mais citados: mudanças de humor, insônia, tristeza e choro incontrolável, dores de cabeça e corporais, sensação de ser incapaz de cuidar do bebê, medo de fazer mal ao bebê, culpa, ansiedade, ataques de pânico e até mesmo ideação suicida^(23)^.

A escuta ativa e qualificada do enfermeiro na atenção à saúde mental desde o pré-natal foi outra prática aperfeiçoada após a educação permanente em saúde, o que indica que a prática de enfermagem se encontra mais humanizada e contínua. Observou-se também melhor colaboração entre os enfermeiros e outros profissionais de saúde na abordagem à mulher com indicativos de DPP, com atenção integral às suas necessidades físicas, emocionais e sociais. A literatura refere que a escuta ativa qualificada pode melhorar o entendimento eficaz das reais necessidades dos usuários e melhor efetividade do cuidado. Além disso, ela permite um cuidado humanizado, fortalecendo o vínculo entre o profissional e usuário, provendo um acolhimento mais efetivo e resolutivo^(24)^.

A visita domiciliar também foi uma atividade aprimorada e é vista na literatura como uma oportunidade de suporte e educação em saúde para as puérperas e seus familiares. Estudos mostram que mulheres que recebem visita domiciliar dos profissionais de saúde têm menor probabilidade de desenvolver sintomas de depressão pós-parto, além de fortalecer e estimular uma melhor interação mãe-bebê, sendo essencial para o bem-estar da mãe e do bebê^(25)^. Além disso, o suporte emocional e apoio social às puérperas são considerados fatores protetores significativos contra a DPP^(26)^. Quando os profissionais realizam as visitas domiciliares, há a oportunidade de rastreamento da DPP nas mulheres que não comparecem à consulta puerperal^(25,27)^.

Outro destaque refere-se ao atendimento multiprofissional, que teve melhora significativa no manejo e fluxo de atendimento às mulheres com DPP. A atuação da equipe multiprofissional é determinante para oferecer o suporte integral necessário que atenda às necessidades físicas e emocionais das mães, por meio de intervenções educativas e psicoterapêuticas, proporcionando o bem-estar tanto materno quanto para o bebê^(12,28, 29, 30, 31)^.

Também outra habilidade que apresentou melhoria foi o apoio e a orientação de saúde, que devem ser claros e sanar as dúvidas da puérpera e seus familiares. Achados semelhantes em outro estudo apontam que os enfermeiros entrevistados acreditam que a educação em saúde é um fator importante para o preparo das mulheres para o pós-parto^(32)^.

Estudos mostram que a capacitação de enfermeiros reflete em um cuidado ao paciente mais holístico e integral, diminuindo barreiras presentes na identificação e manejo da doença por esses profissionais^(33)^. Além disso, a capacitação voltada à saúde mental da mulher permite maior segurança e empoderamento nas práticas realizadas pelos enfermeiros, o que torna a implementação de estratégias de rastreamento e intervenções de enfermagem mais eficazes^(12)^.

Ademais, observou-se uma maior motivação para busca do conhecimento contínuo sobre a temática, além de uma postura mais proativa e otimista sobre o manejo da doença, após a intervenção educativa.

Por fim, a DPP se não tratada repercute claramente na saúde mental materna no puerpério, com repercussões negativas tanto para a mulher como também para a família^(34)^. Diante disso, enfatiza-se a importância da abordagem dessa condição que é um problema de saúde pública. Assim, é fundamental que secretarias de saúde dos municípios desenvolvam programa de capacitação para os profissionais da APS, a fim de subsidiar a melhoria no cuidado ofertado pelo SUS, conforme recomendações da Política Nacional de Educação Permanente em Saúde (Portaria nº 198/GM de 13 de fevereiro de 2004)^(35)^.

O estudo apresenta como limitações a baixa adesão dos enfermeiros, visto que o ambiente virtual ainda é um obstáculo devido à instabilidade na conexão à internet, e também o fato de a pesquisa ter sido realizada apenas com enfermeiros, sem envolver outros profissionais que assistem às puérperas na APS. Todavia, os profissionais que participaram da pesquisa tiveram envolvimento, interesse no tema e intervenção adotados, o que contribui para a qualidade da assistência na APS.

## CONCLUSÃO

À guisa de conclusão, o objetivo proposto pelo estudo foi alcançado. A intervenção educativa sobre DPP provocou um impacto positivo, pois ampliou conhecimentos, habilidades, práticas e, sobretudo, aperfeiçoou as atitudes dos enfermeiros, possibilitando uma assistência mais empática, humanizada e tecnicamente embasada.

Considerando a importância do tema, sugere-se a realização de mais estudos e o desenvolvimento de outras estratégias a fim de engajar os profissionais de saúde e identificar as melhores intervenções e modos de promover a saúde mental e bem-estar das puérperas.

## Data Availability

https://doi.org/10.48331/SCIELODATA.LQRF9N

# Translation: Impacto de una intervención educativa sobre la depresión posparto para enfermeros de atención primaria: estudio cuasi-experimental

**DOI:** 10.1590/1980-220X-REEUSP-2025-0032es

**Authors:** Débora Alves da Silva, Marli Aparecida Reis Coimbra, Lucas Carvalho Santana, Maria Aline Leocádio, Fernanda Bonato Zuffi, Lúcia Aparecida Ferreira

**Affiliations:** 1 Universidade Federal do Triângulo Mineiro, Uberaba, MG, Brasil.; 2 Universidade Federal do Triângulo Mineiro, Hospital de Clínicas, Uberaba, MG, Brazil.

**Keywords:** Enfermeras y Enfermeros, Depresión Posparto, Educación Continua, Atención Primaria de Salud, Enseñanza

## Abstract

**Objetivo:**

Evaluar el impacto de una intervención educativa sobre la asistencia de enfermería a mujeres con indicios de depresión posparto para enfermeros de atención primaria.

**Método:**

Estudio cuasi-experimental, de tipo antes y después, realizado con 14 enfermeros de atención primaria de salud de un municipio que participaron en una intervención educativa sobre la asistencia de enfermería a mujeres con indicios de depresión posparto. Se realizó un análisis cualitativo de los datos antes y después de la intervención, mediante el análisis de contenido temático de Bardin.

**Resultados:**

De la comparación de las respuestas de los contenidos antes y después de la intervención, los enfermeros mostraron un mayor conocimiento sobre la depresión posparto, especialmente en cuanto a los signos y síntomas específicos, y una mejora en el reconocimiento y el manejo del *baby blues*; comenzaron a utilizar escalas para el seguimiento y a monitorizar mejor los síntomas emocionales; empezaron a adoptar prácticas más humanizadas, además de informar de una mayor seguridad en la atención y en la integración multiprofesional.

**Conclusión:**

La intervención tuvo un impacto positivo y amplió los conocimientos, las habilidades y las actitudes de los enfermeros sobre la depresión posparto, lo que contribuyó a una atención más empática e integral. ReBEC: RBR-3hv3hhs.

## INTRODUCCIÓN

La depresión posparto (DPP) es un problema de salud pública que causa un cuadro de profunda tristeza a las mujeres en el puerperio^(1)^. Sus síntomas aparecen entre las primeras cuatro semanas y hasta un año después del parto, y a menudo se confunden con los síntomas comunes del *Baby blues*, una afección transitoria y leve que se produce en las dos primeras semanas del puerperio debido a los cambios hormonales comunes en este período, lo que dificulta el diagnóstico^(1,2)^.

Cuando no es identificada y tratada, la DPP afecta a la salud y el bienestar de la puérpera, además de provocar el desarrollo de posibles problemas conductuales, físicos y emocionales en los niños^(3)^. Además, esta afección desencadena conflictos conyugales y familiares, ya que se trata de un periodo de mayor demanda emocional para la mujer^(4)^.

A nivel mundial, la DPP afecta al 17,22% de las mujeres, siendo los países en desarrollo los que registran las tasas más altas de la enfermedad^(5)^. En Brasil, la prevalencia es del 26%^(6)^. Sin embargo, la pandemia de COVID-19 se ha reflejado a nivel internacional en un aumento de los casos de DPP y ansiedad en las mujeres. Los estudios presentan prevalencias de hasta el 47,2% de depresión y el 40,8% de ansiedad, además del aumento de los índices de ideación suicida^(7,8)^.

Ante este contexto, la Organización Mundial de la Salud (OMS) recomienda el uso de escalas validadas para la detección de la enfermedad por parte de los profesionales de la salud, como la *Escala de Depresión Postnatal de Edimburgo* (*Edinburgh Postnatal Depression Scale* - EPDS) o el *Cuestionario de Salud del Paciente-9* (*Patient Health Questionnaire-9* - PHQ-9). Además, la OMS enfatiza la importancia de abordar el bienestar emocional de las mujeres embarazadas y puérperas en todas las consultas^(9)^.

En esta perspectiva, el enfermero de Atención Primaria de Salud (APS) destaca entre los profesionales en el manejo de la DPP debido a su proximidad con la mujer durante el ciclo gravídico-puerperal, lo que favorece la detección precoz de signos y síntomas indicativos de la enfermedad^(10,11)^. Sin embargo, el conocimiento moderado sobre el tema y otras lagunas hasta entonces desconocidas interfieren en la identificación precoz de la enfermedad. Entre ellas, destacan las limitaciones en las competencias, como: identificación precoz de la DPP, sentimiento de inseguridad; falta de habilidades y lagunas en el conocimiento técnico-científico y en la gestión y asistencia eficaz a la salud mental de las madres^(12,13)^.

Por lo tanto, se percibe la necesidad de intervenciones de educación profesional sobre el DPP para los enfermeros, tanto por la recomendación de la OMS como por la necesidad de intervenciones sobre el tema y por la evidencia de una formación limitada de los enfermeros. El DPP reduce la calidad de vida de la puérpera y del recién nacido, ya que interfiere en el patrón de salud mental deseable. Además, los estudios muestran que las mujeres con DPP tienen un riesgo 6,5 veces mayor de suicidio y una prevalencia de aproximadamente entre el 7% y el 10% de ideas suicidas en el puerperio^(14,15)^. Por lo tanto, el conocimiento y la aplicación de conductas correctas para la identificación y el tratamiento mejoran la prestación de cuidados por parte de los enfermeros, con el fin de garantizar la mejora continua en el desarrollo de competencias (conocimientos, habilidades y actitudes) y la asistencia de calidad y eficacia de las prácticas de enfermería.

Por lo tanto, este estudio tuvo como objetivo evaluar el impacto de una intervención educativa sobre la asistencia de enfermería a mujeres con indicios de depresión posparto para enfermeros de atención primaria de salud.

## MÉTODO

### TIPO DE ESTUDIO

Se trata de un estudio cuasi experimental^(16)^, del tipo antes y después, con análisis mediante un método cualitativo de los datos en la fase previa y posterior a la intervención y adopción del marco teórico a la luz de Philippe Perrenoud^(17)^. Además, la investigación fue registrada y aprobada en la Plataforma REBEC (descrita en la fase de aspectos éticos). Se eligió este diseño con el fin de estandarizar la intervención realizada. Además, esta fue previamente validada por jueces para garantizar el rigor y la calidad metodológica y responder al objetivo propuesto (descrito en la fase de procedimientos para la recolección de datos). Aunque el estudio es de diseño cuasi-experimental, las evaluaciones antes y después de la intervención fueron de carácter cualitativo, ya que se buscó una comprensión amplia y profunda del conocimiento obtenido por los participantes según la teoría^(17)^ adoptada. Además, el uso del análisis cualitativo para evaluar el efecto de la intervención permitió obtener una visión holística del fenómeno^(18)^. Las investigaciones cualitativas son adecuadas cuando se pretende comprender cuestiones específicas que requieren un análisis más detallado y descriptivo^(19)^.

Asimismo, el estudio se guio por las directrices de los *Criterios consolidados para la presentación de informes sobre investigaciones cualitativas* (*Consolidated criteria for reporting qualitative research* - COREQ).

### LUGAR

El estudio se llevó a cabo en las unidades básicas de salud de un municipio del interior de Minas Gerais, Brasil, referencia sanitaria de la macrorregión sanitaria del Triángulo Sur, Minas Gerais, con 53 equipos de la Estrategia de Salud Familiar distribuidos en 29 unidades, siendo la APS la puerta de entrada al Sistema Único de Salud (SUS) y al seguimiento puerperal.

### POBLACIÓN

La población total correspondió a 96 enfermeros de la APS que trabajaban en el municipio durante el período de realización de este estudio, según los datos cuantitativos facilitados por la secretaría municipal de salud.

### CRITERIOS DE SELECCIÓN

Se consideraron criterios de inclusión: enfermeros que trabajaban en la red de Atención Primaria de Salud y que atendían a puérperas. Como criterios de exclusión: estar de vacaciones o ausente de sus actividades laborales durante la realización de la investigación; aquellos que dejaron de participar en alguna de las reuniones de la intervención educativa; o que, a pesar de firmar el Término de Consentimiento Libre y Esclarecido (TCLE), no respondieron alguna de las entrevistas (antes o después de la intervención).

## DEFINICIÓN DE LA MUESTRA

La muestra se definió de manera intencional y no probabilística. De los 96 enfermeros de APS, 32 manifestaron interés en participar en la investigación; de estos, 28 firmaron el TCLE, el Término de Autorización para el uso de Voz y Sonido (TAVS), respondieron al cuestionario semiestructurado y a las preguntas orientadoras (entrevista previa a la intervención). De los 28 profesionales, 6 integraron el grupo piloto (GP) y 22 el grupo de intervención (GI). De los 22 participantes del GI, 1 enfermero se ausentó por licencia médica; 6 faltaron al menos un día de la intervención; y 1 no participó en la entrevista post-intervención. Así, la muestra final fue de 14 profesionales.

## PROCEDIMIENTOS PARA LA RECOLECCIÓN DE DATOS

Los contactos de los enfermeros se solicitaron a la Sección de Educación en Salud de la Secretaría Municipal de Salud del municipio, y la carta de invitación se envió por teléfono y/o *Whatsapp* ^®^, durante los meses de septiembre y octubre de 2023. La recolección de datos, antes y después de la intervención, se realizó de forma remota (a través de la plataforma *Google Meet*) en las fases pre-intervención, entre noviembre y diciembre de 2023, y post-intervención, en abril de 2024, o hasta que se completaran 30 días después de la intervención.

Las entrevistas se realizaron de forma individual y los testimonios se recopilaron en un dispositivo de grabación de audio independiente, sin que se realizara ninguna grabación a través de la plataforma. Por último, para evitar el riesgo de una posible pérdida de la confidencialidad de los datos recopilados, los participantes fueron codificados con la letra «E», que significa «Entrevistado», seguida de un número arábigo que hace referencia a la secuencia de las entrevistas, de E01 a E14.

Para la recolección de datos, antes y después, se utilizó un cuestionario semiestructurado elaborado por los autores, cuyo objetivo fue evaluar los conocimientos, habilidades y actitudes de los enfermeros de APS en cuanto a la asistencia de enfermería a mujeres con indicios de DPP. El contenido de los instrumentos de recolección de datos y el guion de la intervención educativa fueron validados cualitativamente por tres jueces^(20)^ seleccionados mediante análisis del currículo Lattes, todos ellos enfermeros, doctores, especialistas en enfermería, salud mental y/o salud de la mujer.

El instrumento constaba de dos partes, la primera basada en datos «sociodemográficos y profesionales»: sexo, edad, tiempo de formación como enfermero, tiempo de actuación en atención primaria, cursos y/o especialización específica en salud mental; y si la secretaría municipal de salud ofrecía educación permanente orientada a la atención de enfermeros a mujeres con indicios de depresión posparto. La segunda parte se compuso de preguntas orientadoras según el marco teórico adoptado^(17)^: «¿Qué sabe hoy sobre la depresión posparto?; ¿Cómo busca hoy información sobre el tema?; ¿Cómo identifica hoy a una mujer con indicios de depresión posparto?; ¿Qué cuidados de enfermería considera importantes hoy en día para la asistencia de enfermería a mujeres con indicios de depresión posparto?; ¿Percibe hoy en día un movimiento para la atención a mujeres con indicios de depresión posparto en el ámbito municipal? ¿O es algo nuevo, sin compromiso por parte de los profesionales? ¿Hay apertura para tratar el tema?».

## INTERVENCIÓN EDUCATIVA

La planificación y ejecución de la intervención educativa se basaron en la teoría de las competencias de Philippe Perrenoud^(17)^. Según este sociólogo, desarrollar competencias profesionales es capacitar a los individuos para actuar de manera eficaz en diferentes situaciones de la vida cotidiana, sabiendo articular los conocimientos, las habilidades y las actitudes en la solución de diversos problemas^(17)^.

Basada en este marco teórico, la intervención educativa tuvo como contenido teórico-práctico sobre la DPP: la llegada de un nuevo bebé y los cambios en el contexto familiar; conceptos de DPP y Baby blues; epidemiología; fisiopatología, signos y síntomas de la enfermedad; desenlaces graves; impacto de la DPP en la salud de la madre, el bebé y el núcleo familiar; políticas públicas de salud orientadas a la salud mental materna; detección de la DPP con escalas validadas; red de apoyo a la mujer con DPP; competencias del enfermero; y flujo de atención a la mujer con DPP en el municipio estudiado. Los temas abordados siguieron la teoría propuesta^(17)^, con énfasis en los conceptos, las prácticas y los contextos para innovar y mejorar las competencias profesionales.

Además, para alcanzar la teoría básica^(17)^, se presentaron situaciones basadas en las experiencias y prácticas diarias de enfermería de los enfermeros de APS, compartiendo experiencias entre los profesionales, estimulándolos siempre a participar y construir conjuntamente el proceso de aprendizaje.

Además, las conferencias y debates se planificaron e implementaron con el objetivo de estimular la aplicación de los conocimientos adquiridos en las diversas situaciones prácticas y contextos variados que podrían darse durante la atención de enfermería a mujeres con indicios de DPP en las unidades básicas de salud. Asimismo, se estimuló la autoevaluación de los enfermeros sobre la atención prestada y lo recomendado en la literatura, con el fin de estimular la reflexión sobre sus competencias, impulsando así la mejora continua de las competencias profesionales^(17)^.

La intervención educativa fue dirigida por cuatro facilitadoras, tres enfermeras y una psicóloga, profesionales con maestría, doctorado, experiencia y especialistas en el área de salud mental y/o salud de la mujer, pertenecientes al grupo de investigación de la universidad federal del municipio.

La intervención educativa se llevó a cabo, de forma colectiva, en cuatro encuentros de dos horas de duración cada uno (de 14:00 a 16:00), durante el mes de marzo de 2024, dos de ellos presenciales (sala de reuniones de la secretaría municipal de salud del municipio) y dos a distancia (plataforma *Google Meet*)*,* con un intervalo de dos días entre el primer y el segundo día de intervención, de cinco días entre el segundo y el tercer día, y de dos días entre el tercero y el cuarto. Se utilizaron presentaciones de diapositivas en Power Point, dinámicas y computadora. Las competencias de los participantes en la investigación se evaluaron en la fase previa y posterior a la intervención mediante un guion de análisis cualitativo (preguntas orientativas) previamente validado y evaluado en un estudio piloto.

## ANÁLISIS Y TRATAMIENTO DE LOS DATOS

Los datos sociodemográficos y profesionales se almacenaron en una hoja de cálculo de *Excel*®, se exportaron al *Statistical Package for the Social Sciences* (SPSS) (versión 29) y se realizó un análisis estadístico descriptivo simple. Para el análisis de los testimonios, estos se transcribieron íntegramente en un documento de *Microsoft Word* ^®^ y se analizaron cualitativamente, basándose en el análisis de contenido temático de Laurence Bardin, que se basa en tres etapas: 1- preanálisis, 2- exploración del material, 3- tratamiento de los resultados obtenidos e interpretación^(21)^. Los testimonios se organizaron, codificaron y categorizaron con el apoyo del software ATLAS.ti^®^, versión 24.

## ASPECTOS ÉTICOS

El estudio fue aprobado por el Comité de Ética y Investigación, de conformidad con el CAAE n.º 72614123.7.0000.5154 y el dictamen 6.278.334/2023, y registrado en la plataforma del Registro Brasileño de Ensayos Clínicos (ReBEC) con el número de registro: RBR-3hv3hhs. Asimismo, se llevó a cabo de conformidad con la Resolución 466/2012, la Resolución n.º 510, de 7 de abril de 2016, y la Circular n.º 2/2021/CONEP/SECNS/MS. Además, a quienes aceptaron participar en el estudio se les solicitó su consentimiento mediante la firma del TCLE y, para garantizar el anonimato, los participantes fueron identificados mediante un código numérico (ENT 01, ENT 02, ENT 03...), donde ENT significa «Entrevistado», respetando la confidencialidad de los nombres.

## RESULTADOS

Catorce enfermeros participaron en la investigación, siendo el 85,7% (12) de sexo femenino, con una edad predominante de entre 31 y 40 años (71,4%) y una antigüedad en el cargo de entre 12 y 16 años (49,9%). El curso de especialización en salud mental representó el 21,4%. No se informó de cursos realizados por la Secretaría de Salud. Lo Cuadro 1 muestra las características sociodemográficas y profesionales investigadas.

**Cuadro 1:** Características sociodemográficas y profesionales de los participantes Características sociodemográficas y profesionales de los participantes - Uberaba, MG, Brasil, 2025.

Variables	n (n= 14)	%
**Sexo**		
Mujer	12	85,7
Hombre	2	14,3
**Grupo de edad**		
Hasta 30 años	1	7,2
31 - 40	10	71,4
41 - 51	3	21,4
**Duración del servicio**		
De 6 a 9 años	12	85,7
15 años o +	2	14,3
**Período de formación**		
De 6 a 9 años	5	35,7
De 12 a 16 años	7	50,0
25 a 27 años	2	14,3
**Cursos de postgrado en salud mental**		
Sí	3	21,4
No	11	78,6
**Curso de formación impartido por Secretaría de Salud**		
Sí	0	0,0
No	14	100

Fuente: Elaboración propia, 2025.

## FASE PRE-INTERVENCIÓN

En la fase previa a la intervención, se evaluaron las competencias previas de los enfermeros de APS en la asistencia de enfermería a mujeres con indicios de DPP a la luz de la teoría de las competencias de Philippe Perrenoud^(17)^. De la primera evaluación, previa a la intervención, pero centrada en la teoría propuesta^(17)^, surgieron tres categorías que demostraron la aplicabilidad de la teoría^(17)^ al estudio: Conocimientos de los enfermeros sobre la DPP, Habilidades de los enfermeros en la asistencia a mujeres con indicios de DPP y Actitudes de los enfermeros en la asistencia a mujeres con indicios de DPP.

## CONOCIMIENTO DE LOS ENFERMEROS SOBRE LA DPP

A priori, los entrevistados podían identificar algunos indicios de DPP basados en el «comportamiento materno», entre los que destacaban como principales comportamientos observados: problemas con la lactancia, desatención de la madre, percepción de menor vínculo madre-bebé, silencio de la puérpera, mirada desmotivada y presencia de ojeras. Sin embargo, otras situaciones de la enfermedad eran menos comprendidas o enfatizadas por ellos.

*Si tiene síntomas de tristeza, ¿no? De depresión, ansiedad, si tiene... ¿cómo está ese vínculo entre la madre y el bebé?* (E04)

*Si ella está logrando amamantar a su recién nacido, amamantar a su hijo y eso, eso es un factor, si ella no ha logrado amamantar* (E08)

*[...] a veces la madre, muy desmotivada, con la mirada perdida, sin reacción [...] la madre está allí totalmente desconectada [...]* (E12)

*[...] puede haber alteraciones en los patrones de sueño, en la alimentación, un cambio en los sentimientos hacia el niño, puede perder interés por las actividades que le gustaban, alteraciones emocionales, irritabilidad, llanto excesivo, ¿no? [...]* (E07)

Asimismo, los enfermeros informaron de un conocimiento limitado y una falta de formación específica sobre la DPP, además de que la búsqueda de conocimientos se produce principalmente ante una necesidad inmediata, lo que conlleva una actitud reactiva en la atención del profesional. Se observan otros temas infravalorados por los enfermeros, como «Cambios hormonales», «Baby blues» y «Consecuencias de la depresión posparto», lo que indica un enfoque limitado en estos temas.

*Sé muy poco [...] cuando atiendo a alguna paciente, entonces busco información. ¿Entiendes?* (E01)

*Nunca he atendido a una paciente con depresión posparto aquí en la unidad, ¿verdad?* (E05)

*Específicamente sobre la depresión posparto sé muy poco, ¿verdad? Sabemos un poco sobre los síntomas depresivos, ¿verdad? (E11)*

*Para ser sincera, normalmente busco información cuando tengo alguna paciente aquí, ¿no? (E12)*

*[…] porque hoy en día existe ese otro síndrome, ¿no?, del que hablan, no sé si se llama Blue Baby, ¿no? que es más pasajero, ¿no?* (E12)

*A veces las mujeres intentan suicidarse. He oído algunos casos.* (E01)

## HABILIDADES DE LOS ENFERMEROS EN LA ASISTENCIA DE ENFERMERÍA A MUJERES CON INDICIOS DE DPP

Se identificaron precozmente, en los testimonios, signos y síntomas en las consultas rutinarias del enfermero y otros profesionales, así como la percepción de la importancia de la atención multiprofesional, la escucha activa, la derivación y la acogida de la paciente.

*Cuando vuelve para la prueba del talón, siempre hablo con ella y le pregunto cómo está. ¿Cómo va la lactancia? Voy hablando con ella. Hay algunas que veo que están bien.* (E03)

*Con nosotros en el seguimiento, a veces en la sala de vacunas [...] Entonces, identificamos cuando esa mujer viene a la unidad, acabamos hablando, ¿no? Saber identificar y derivar, cuando es necesario.* (E07)

*[...] concertamos una cita con un médico de familia para orientarla si necesita acudir a un psiquiatra, si puede seguir con el seguimiento aquí con nosotros.* (E07)

*Y así, creo que nuestra herramienta de trabajo es escuchar, acoger, creo que es lo fundamental, lo principal.* (E13)

También se identificó la importancia de la colaboración entre el equipo de salud y los familiares de las puérperas en la percepción de los signos y síntomas del DPP. Además, en las declaraciones se percibe la importancia de la búsqueda activa realizada por los agentes comunitarios de salud a partir de las visitas domiciliarias, la necesidad de capacitar a los equipos sobre el tema y la falta de utilización de escalas específicas para la detección de síntomas de DPP que podrían ayudar en la atención. El vínculo de la paciente con el equipo de salud también se mencionó como una estrategia que permite la identificación y el manejo de la DPP.

*Entonces es más cuando la madre o la familia nos buscan. Para hablar de algún síntoma que la madre está teniendo.* (E09)

*Porque en el momento en que vas a hacer la visita, nosotros {el equipo} tenemos una mirada diferente, pero a veces puede pasar desapercibido, y entonces orientamos a la familia en el sentido de que, si es necesario, si se presenta algún cambio, intenten acudir al centro.* (E14)

*Entonces, el agente comunitario de salud a veces nos trae algo relacionado con el hogar, el apoyo familiar, así que es más en esas situaciones.* (E12)

*[...] hacer una formación continua con los agentes comunitarios para que ellos también estén atentos.* (E09)

*[...] capacitar al equipo [...]* (E07)

*Hoy en día no utilizo ninguna escala en la consulta, ninguna escala de salud mental [...]* (E11)

*Buscar tener un vínculo con esa gestante durante todo el prenatal [...]* (E05)

*Creo que el principal cuidado es el seguimiento constante* (E05)

## ACTITUDES DE LOS ENFERMEROS PARA LA ASISTENCIA DE ENFERMERÍA A MUJERES CON INDICIOS DE DPP

Dentro de esta categoría principal surgieron los temas: «Apertura a la capacitación», «Conocer el flujo de atención», «Deseo de mejorar la asistencia» e «Interés del equipo sobre el tema». Se percibió la apertura de los enfermeros a actividades de capacitación sobre el tema.

*[...] creo que sí hay apertura para tratar el tema, porque veo que formamos parte de un equipo al que le gusta mucho aprender y actualizarse; sin embargo, es un tema con muy poca incidencia [...]* (E04)

*Mira, creo que hay apertura. Los profesionales aquí [...] están muy abiertos a todo tipo de capacitaciones [...]* (E06)

## FASE POST-INTERVENCIÓN

En la fase post-intervención, se evaluó el impacto de la intervención educativa con el mismo guion de la fase pre-intervención (según el marco teórico adoptado) en las competencias de los enfermeros de APS en la asistencia de enfermería a mujeres con indicios de depresión posparto. En esta etapa se identificaron las tres categorías a priori identificadas en la fase previa a la intervención: Conocimientos de los enfermeros sobre la depresión posparto; Habilidades de los enfermeros en la asistencia de enfermería a mujeres con indicios de depresión posparto; Actitudes de los enfermeros en la asistencia de enfermería a mujeres con indicios de depresión posparto.

## CONOCIMIENTOS DE LOS ENFERMEROS SOBRE LA DPP

Esta categoría expresa la mejora del conocimiento sobre la DPP y la percepción de la importancia del uso de una escala para la detección de la DPP. Además, los enfermeros lograron percibir la prevalencia de la DPP como un problema y diferenciarla del *baby blues*.

*[...] podemos utilizar las escalas, hay algunas escalas que ya están validadas, que podemos utilizar para detectar entre nuestras puérperas si alguna tiene indicios de depresión posparto [...]* (E03)

*[...] teóricamente, sé que tenemos la escala que nos presentaron, la de depresión posparto, que es la de {Edimburgo} [...]* (E13)

*Bueno, sé que {el DPP} está mucho más presente de lo que imaginaba.* (E02)

*[...] observar si los síntomas solo se presentan en los primeros días y desaparecen o si persisten, para que podamos caracterizarlo como una depresión [...]* (E10)

*[...] logré diferenciar el baby blues de la depresión. La depresión posparto es un poco más grave. Puede durar desde meses hasta dos años después del parto [...]* (E12)

Después de la capacitación, los enfermeros comprendieron mejor la gravedad del problema y la importancia de identificar los signos y síntomas de la DPP. Asimismo, también percibieron mejor la infravaloración y la infradeclaración de la enfermedad.

*[...] hoy conozco el cuadro clínico y presto más atención después de haber recibido la formación, presto más atención a lo que me cuenta la paciente [...]* (E14)

*Hoy, después del curso, tenemos un amplio conocimiento sobre los signos y síntomas [...] Y ahora podemos visualizar, puedo visualizar los síntomas durante la consulta posparto* (E08)

*Y es una enfermedad que muchas veces pasa desapercibida, se confunde mucho con la sobrecarga materna y a veces no le damos tanta importancia.* (E03)

*[...] era un tema muy poco notificado, no teníamos mucha información, no era una preocupación de los enfermeros, del ginecólogo que atiende [...]* (E08)

También se observó la concienciación de los profesionales sobre los graves desenlaces de la DPP, además de la universalidad de la enfermedad.

*[...] Llegar al punto de una depresión más grave, llegar al punto de un intento de suicidio o incluso de agresión al bebé.* (E04)

*[...] más graves, que pueden causar mayores daños tanto a la puérpera como al bebé [...]* (E13)

## HABILIDADES DE LOS ENFERMEROS EN LA ASISTENCIA DE ENFERMERÍA A MUJERES CON INDICIOS DE DPP

Habilidades como la identificación de señales de alerta indicativas de DPP, la identificación precoz de los signos y síntomas, la conciencia de la importancia de la atención multiprofesional, la escucha activa y cualificada de la paciente y la visita domiciliaria fueron temas que surgieron en los testimonios de los profesionales.

*Las mujeres suelen presentar signos de tristeza. A veces, durante el embarazo, ya se nota, a veces, algunos signos al final del embarazo [...] Solemos identificarlas justo después del parto, entonces hay un período. Las mujeres a menudo comienzan a dar alguna señal durante el prenatal [...]* (E10)

*[...] cómo duerme; cuestiones relacionadas con la lactancia, si tiene dificultades, si le duele al amamantar; también es muy importante que sepamos cómo está su autoestima, porque cuando nace el bebé, toda la atención se centra en él [...] observando estas cuestiones, cómo están sus emociones, si tiene episodios de tristeza profunda, de llanto, de pensamientos intrusivos, eso.* (E13)

*Y lo que no está a nuestro alcance, lo compartimos con el equipo.* (E07)

*[...] también organizar una visita a esa mujer, para no dejarla totalmente sin atención profesional durante muchos días, precisamente porque va a necesitar un seguimiento* más *cercano [...]* (E05)

Los enfermeros también mencionaron la importancia de ampliar la mirada hacia la puérpera, observándola de manera integral.

*[...] realmente necesitamos ampliar esta mirada para ver a la puérpera, para también orientarla [...] cuando llega para llevar al niño a hacer el test de Galén, primero trato de atenderla yo mismo, de manera integral* (E01)

*[...] después de la formación, presto más atención a lo que me cuenta la paciente.* (E14)

El apoyo y la orientación sanitaria, así como la monitorización continua de los signos y síntomas, la humanización de la asistencia y el apoyo de la enfermería, se mencionaron como factores esenciales en la atención a la puérpera.

*[...] y nosotros, como enfermeros, seguimos acompañando a esta puérpera en sus dificultades, con orientaciones [...]* (E13)

*[...] hemos intentado programarle una consulta al menos cada 15 días, además de la atención semanal con la psicóloga.* (E14)

*[...] creo que el principal cuidado sería la humanización, para que entendamos que esa mujer está pasando por un momento delicado del posparto [...]* (E08)

## ACTITUDES DE LOS ENFERMEROS EN LA ASISTENCIA DE ENFERMERÍA A MUJERES CON INDICIOS DE DPP

En los testimonios se presentaron actitudes como la búsqueda activa de conocimientos y la motivación del profesional para mejorar.

*[...] hoy, con la formación, ha sido bueno que se haya orientado, se hayan aportado ejemplos, cosas nuevas, nuevas bibliografías [...]* (E01)

*[...] creo que hacía mucho tiempo que no teníamos una formación tan válida con profesionales tan comprometidos; ha sido muy buena, muy rica* (E06)

*Entonces, creo que es algo que tenemos que ir mejorando, cambiando. Creo que he mejorado en eso, en esa cuestión.* (E01)

También se mencionaron el optimismo de los enfermeros, los debates sobre el tema y el aumento de la seguridad en la atención a las puérperas.

*Lo discutimos mucho y hoy creo que lo intento, he cambiado mi forma de ver las cosas.* (E01)

*Así que me siento más empoderada para atender a estas mujeres, sin duda, a estas puérperas.* (E07)

*[...] después de la formación que recibimos, me sentí mucho más segura con respecto al tema [...]* (E12)

## COMPARACIÓN DE LOS CONOCIMIENTOS, HABILIDADES Y ACTITUDES ANTES Y DESPUÉS DE LA INTERVENCIÓN

El análisis comparativo del momento previo y posterior a la intervención, presentado en el Cuadro 2, evidencia que hubo un impacto positivo en los conocimientos, habilidades y actitudes de los enfermeros para actuar ante la DPP.

**Cuadro 2:** Comparación de conocimientos, competencias y actitudes antes y después de la intervención - Uberaba, MG, Brasil, 2025.

Competencia	Pre-intervención	Post-intervención
**Conocimientos**	Conocimiento de los indicios de DPP basado en el comportamiento materno. Informes de falta de información.	Ampliación de los conocimientos sobre la DPP (signos y síntomas) y uso de escalas de detección. Conocimiento de la prevalencia y gravedad clínica de la enfermedad en.
**Habilidades**	Toma de conciencia de la necesidad de una identificación precoz de los signos y síntomas, de la importancia del equipo multiprofesional y de la necesidad de formación.	Identificación de los signos de alarma. Comprensión de la importancia del equipo multiprofesional, de la escucha activa y cualificada y de las visitas a domicilio. Ampliación de la perspectiva, comprensión del seguimiento y de los cuidados humanizados.
**Actitudes**	Apertura a la formación. Deseo de mejorar los cuidados.	Comprensión a través de la búsqueda de conocimientos, motivación, aumento de la seguridad en el cuidado de la puérpera.

Fuente: Elaboración propia, 2025.

## DISCUSIÓN

Al relacionar la teoría de Perrenoud^(17)^ y el tema de la investigación, se observa que las competencias profesionales favorecen la resolución de diversos contratiempos en las actividades cotidianas. Durante el desempeño de sus actividades, los enfermeros necesitan articular la práctica con los conocimientos, las habilidades y las actitudes adquiridas. Este hecho fue favorable en la evaluación de esta investigación.

La intervención educativa tuvo un impacto positivo en los conocimientos, habilidades y actitudes de los enfermeros de APS a la luz del marco teórico^(17)^ adoptado. Las diferencias en los análisis de los contenidos en los momentos previos y posteriores a la intervención ponen de manifiesto las deficiencias en la actualización y/o el conocimiento sobre el tema antes de la intervención y el compromiso y el desarrollo de habilidades después de la misma.

Tras la intervención educativa, los resultados demostraron satisfactorias mejoras relacionadas con las competencias de los enfermeros en el abordaje de las mujeres con DPP.

La capacitación mejoró los niveles de comprensión sobre el tema, con una mejor comprensión de las escalas y cómo utilizarlas para la identificación precoz de la DPP. Esto concuerda con un estudio^(22)^ que evaluó el efecto de una capacitación sobre la DPP para enfermeras con el fin de determinar su valor en la práctica de la enfermería. Los resultados mostraron que hubo un aumento significativo del conocimiento y la capacidad de proporcionar educación en salud sobre el DPP a las madres, en comparación con los resultados analizados en las pruebas previas y posteriores.

Además, la intervención educativa propició una mejora en las habilidades técnicas de los enfermeros en relación con la asistencia de enfermería a las mujeres con indicios de DPP. Al centrar la atención en los signos y síntomas de alerta de la enfermedad, los enfermeros mencionaron tristeza profunda, baja autoestima, desinterés por el autocuidado, aislamiento, desinterés por el cuidado del bebé y la lactancia, falta de interacción social y familiar, trastornos del sueño, llanto fácil y pensamientos intrusivos. En un estudio de revisión se encontraron síntomas similares, siendo los más citados: cambios de humor, insomnio, tristeza y llanto incontrolable, dolores de cabeza y corporales, sensación de ser incapaz de cuidar al bebé, miedo de hacerle daño, culpa, ansiedad, ataques de pánico e incluso ideas suicidas^(23)^.

La escucha activa y cualificada del enfermero en la atención a la salud mental desde la etapa prenatal fue otra práctica perfeccionada tras la educación permanente en salud, lo que indica que la práctica de la enfermería es más humanizada y continua. También se observó una mejor colaboración entre los enfermeros y otros profesionales de la salud en el abordaje de las mujeres con indicios de DPP, con atención integral a sus necesidades físicas, emocionales y sociales. La literatura señala que la escucha activa cualificada puede mejorar la comprensión eficaz de las necesidades reales de los usuarios y la eficacia de la atención. Además, permite una atención humanizada, fortaleciendo el vínculo entre el profesional y el usuario, proporcionando una acogida más eficaz y resolutiva^(24)^.

La visita domiciliaria también ha sido una actividad mejorada y se considera en la literatura como una oportunidad de apoyo y educación en salud para las puérperas y sus familiares. Los estudios demuestran que las mujeres que reciben visitas domiciliarias de profesionales de la salud tienen menos probabilidades de desarrollar síntomas de depresión posparto, además de fortalecer y estimular una mejor interacción entre la madre y el bebé, lo cual es esencial para el bienestar de ambos^(25)^. Además, el apoyo emocional y social a las mujeres en el puerperio se consideran factores protectores significativos contra la DPP^(26)^. Cuando los profesionales realizan visitas domiciliarias, existe la oportunidad de detectar el TPP en las mujeres que no acuden a la consulta posparto^(25,27)^.

Otro aspecto destacado es la atención multiprofesional, que ha mejorado significativamente en el manejo y el flujo de la atención a las mujeres con TPP. La actuación del equipo multidisciplinar es determinante para ofrecer el apoyo integral necesario que satisfaga las necesidades físicas y emocionales de las madres, mediante intervenciones educativas y psicoterapéuticas, proporcionando el bienestar tanto de la madre como del bebé^(12,28, 29, 30, 31)^.

Otra habilidad que mejoró fue el apoyo y la orientación en materia de salud, que deben ser claros y resolver las dudas de la puérpera y sus familiares. Hallazgos similares en otro estudio señalan que los enfermeros entrevistados creen que la educación en salud es un factor importante para la preparación de las mujeres para el posparto^(32)^.

Los estudios demuestran que la formación de los enfermeros se refleja en una atención al paciente más holística e integral, reduciendo las barreras presentes en la identificación y el manejo de la enfermedad por parte de estos profesionales^(33)^. Además, la capacitación orientada a la salud mental de la mujer permite una mayor seguridad y empoderamiento en las prácticas realizadas por los enfermeros, lo que hace que la implementación de estrategias de detección e intervenciones de enfermería sea más eficaz^(12)^.

Asimismo, se observó una mayor motivación para la búsqueda de conocimientos continuos sobre el tema, además de una actitud más proactiva y optimista sobre el manejo de la enfermedad, tras la intervención educativa.

Por último, la DPP, si no se trata, repercute claramente en la salud mental materna en el puerperio, con repercusiones negativas tanto para la mujer como para la familia^(34)^. Ante esto, se enfatiza la importancia de abordar esta condición, que es un problema de salud pública. Por lo tanto, es fundamental que las secretarías de salud de los municipios desarrollen programas de capacitación para los profesionales de la APS, con el fin de subsidiar la mejora en la atención ofrecida por el SUS, de acuerdo con las recomendaciones de la Política Nacional de Educación Permanente en Salud (Portaria nº 198/GM de 13 de febrero de 2004)^(35)^.

El estudio presenta como limitaciones la baja adhesión de los enfermeros, ya que el entorno virtual sigue siendo un obstáculo debido a la inestabilidad de la conexión a Internet, y también el hecho de que la investigación se haya realizado solo con enfermeros, sin involucrar a otros profesionales que atienden a las puérperas en la APS. Sin embargo, los profesionales que participaron en la investigación mostraron implicación e interés en el tema y en la intervención adoptada, lo que contribuye a la calidad de la asistencia en la APS.

## CONCLUSIÓN

A modo de conclusión, se ha alcanzado el objetivo propuesto por el estudio. La intervención educativa sobre el DPP tuvo un impacto positivo, ya que amplió los conocimientos, las habilidades y las prácticas y, sobre todo, mejoró las actitudes de los enfermeros, lo que permitió una asistencia más empática, humanizada y con una base técnica.

Teniendo en cuenta la importancia del tema, se sugiere la realización de más estudios y el desarrollo de otras estrategias con el fin de involucrar a los profesionales de la salud e identificar las mejores intervenciones y formas de promover la salud mental y el bienestar de las puérperas.

## Data Availability

https://doi.org/10.48331/SCIELODATA.LQRF9N
