# Supplementary material for: ERRATUM: Impact of an educational intervention on postpartum depression for primary care nurses: a quasi-experimental study
Source: Rev Esc Enferm USP. 2025 Dec 1;59:e20250032er. doi: 10.1590/1980-220X-REEUSP-2025-0032eren (PMC12679712; doi:10.1590/1980-220X-REEUSP-2025-0032eren)
Supplement: Related texts

# Correction: ERRATA: Impacto de uma intervenção educativa sobre depressão pós-parto para enfermeiros da atenção primária: estudo quase-experimental

No artigo “Impacto de uma intervenção educativa sobre depressão pós-parto para enfermeiros da atenção primária: estudo quase-experimental”, com número de DOI: https://doi.org/10.1590/1980-220X-REEUSP-2025-0032pt, publicado no periódico Revista da Escola de Enfermagem da USP, 59:e20250032, 2025, na página 1:

**Onde se lia:**

**Autor correspondente:**

Débora Alves da Silva

Rua Lourival Pereira Gomes, 570, 

Parque das Laranjeiras

04764-070 – Uberaba, MG, Brasil

dalvesenf@gmail.com

**Leia-se:**

**Autor correspondente:**

Débora Alves da Silva

Av. Getúlio Guaritá, 107, Nossa Sra. da Abadia

38025-440 – Uberaba, MG, Brasil

dalvesenf@gmail.com

Profa. Dra. Maria Amélia de Campos Oliveira

*Editora-chefe*

# Correction: ERRATA: Impacto de una intervención educativa sobre la depresión posparto para enfermeros de atención primaria: estudio cuasi-experimental

En el artículo “Impacto de una intervención educativa sobre la depresión posparto para enfermeros de atención primaria: estudio cuasi-experimental”, con número de DOI: https://doi.org/10.1590/1980-220X-REEUSP-2025-0032es, publicado en la revista Revista da Escola de Enfermagem da USP, 59:e20250032, 2025, en la página 1:

**Donde se leía:**

**Autor correspondiente:**

Débora Alves da Silva 

Rua Lourival Pereira Gomes, 570, Parque das Laranjeiras 

04764-070 – Uberaba, MG, Brasil 

dalvesenf@gmail.com

**Lea:**

**Autor correspondiente:**

Débora Alves da Silva

Av. Getúlio Guaritá, 107, Nossa Sra. da Abadia

38025-440 – Uberaba, MG, Brasil

dalvesenf@gmail.com

Prof. Dra. Maria Amélia de Campos Oliveira

*Editora in jefe*
